# Tracing the Evolution of Lineage-Specific Transcription Factor Binding Sites in a Birth-Death Framework

**DOI:** 10.1371/journal.pcbi.1003771

**Published:** 2014-08-21

**Authors:** Ken Daigoro Yokoyama, Yang Zhang, Jian Ma

**Affiliations:** 1 Institute for Genomic Biology, University of Illinois at Urbana-Champaign, Urbana, Illinois, United States of America; 2 Department of Bioengineering, University of Illinois at Urbana-Champaign, Urbana, Illinois, United States of America; University of California, San Diego, United States of Ameirca

## Abstract

Changes in cis-regulatory element composition that result in novel patterns of gene expression are thought to be a major contributor to the evolution of lineage-specific traits. Although transcription factor binding events show substantial variation across species, most computational approaches to study regulatory elements focus primarily upon highly conserved sites, and rely heavily upon multiple sequence alignments. However, sequence conservation based approaches have limited ability to detect lineage-specific elements that could contribute to species-specific traits. In this paper, we describe a novel framework that utilizes a birth-death model to trace the evolution of lineage-specific binding sites without relying on detailed base-by-base cross-species alignments. Our model was applied to analyze the evolution of binding sites based on the ChIP-seq data for six transcription factors (GATA1, SOX2, CTCF, MYC, MAX, ETS1) along the lineage toward human after human-mouse common ancestor. We estimate that a substantial fraction of binding sites (∼58–79% for each factor) in humans have origins since the divergence with mouse. Over 15% of all binding sites are unique to hominids. Such elements are often enriched near genes associated with specific pathways, and harbor more common SNPs than older binding sites in the human genome. These results support the ability of our method to identify lineage-specific regulatory elements and help understand their roles in shaping variation in gene regulation across species.

## Author Summary

Recent experimental studies showed that the evolution of transcription factor binding sites (TFBS) is highly dynamic, with sites differing a great deal even between closely related mammalian species. Despite the substantial experimental evidence for rapid divergence of regulatory protein-binding events across species, computational methods designed to analyze regulatory elements evolution have focused primarily on phylogenetic footprinting approaches, in which putative functional regulatory elements are identified according to strong sequence conservation. Cross-species comparisons of non-coding sequences are limited in their ability to fully understand the evolution of regulatory sequences, particularly in cases where the elements are selected for novelty or species-specific. We have developed a novel framework to reconstruct the history of lineage-specific TFBS and showed that large amount of TFBS in human were born after human-mouse divergence. These elements also have distinct biological implications as compared to more ancient ones. This method can help understand the roles of lineage-specific TFBS in shaping gene regulation across different species.

## Introduction

Changes in gene regulation play a key role in the evolution of morphological traits [1]–[3]. At the level of transcription, gene expression is controlled via transcription factor (TF) proteins that selectively bind to cis-regulatory elements in a sequence-specific manner [2], [4]. Utilizing chromatin immunoprecipitation of specific TFs followed by high-throughput sequencing (ChIP-seq), recent studies showed that the evolution of these transcription factor binding sites (TFBS) is highly dynamic, with sites differing a great deal even within mammals [5]–[9].

Despite substantial experimental evidence for rapid divergence of regulatory protein-binding events across species, computational models designed to analyze regulatory elements using cross-species comparisons have focused primarily upon ‘phylogenetic footprinting’ approaches, in which putatively functional regulatory elements are identified according to sequence conservation [10]–[15]. Previous computational studies have inferred the evolution of regulatory elements using, for example, the emergence of new conserved elements specific to a particular clade in the phylogeny [16] or lineage-specific alterations leading to a loss-of-function phenotype [17], [18]. Although such approaches have been helpful in understanding lineage-specific regulatory element evolution, all inherently rely upon fixed cross-species alignments, which are frequently of low quality within non-coding regions in the genome [19]–[21]. Previous studies have estimated that more than 15% of aligned bases within human-mouse whole-genome alignments are incorrect [22] and the error rate increases when more species are involved [19]. Ancestral reconstruction, which is sensitive to details of the multiple alignment, is a particularly challenging problem for non-coding regions [23], [24]. As a consequence, cross-species comparisons of non-coding sequences are limited in their ability to study regulatory sequence evolution, particularly in cases where the elements are selected for novelty or newly-derived. Such newly-derived regulatory elements are not rare; indeed, analyses using human population variation data from the 1000 Genomes Project [25] have shown that human genomic locations under selection undergo considerable turnover and frequently lie outside mammalian-conserved regions [26]. Yet, systematic identification of binding sites for specific TFs and assessment of their conservation and prevalence using cross-species comparisons remains a challenging problem.

In this work, we introduce a novel evolutionary framework through which lineage-specific TFBSs can be inferred on a genome-wide scale. In contrast to conservation-based approaches [13], [16], [27], we utilize a birth-death model to infer ancestral states of a given motif without the use of the base-by-base alignment details in the underlying cross-species sequence alignment. Gains and losses of TFBS have been explicitly used both to improve cross-species sequence comparisons and to detect cis-regulatory modules, although such models are usually framed within the context of an alignment [21], [28]. A more similar alignment-free model was previously used to measure the overall rate of TFBS creation along different lineages [29]. In this work, we instead applied our framework to infer lineage-specific TFBS, estimating the branch of origin of each individual TFBS for six TFs. We then studied patterns for TFBS with different branches of origin, including target genes of the newly-derived sites, relationship with within-human variation, overlap with transposable elements, and cell-type specificity of TF-binding. Our results provide strong support that this novel method can help identify lineage-specific regulatory elements, a first step towards understanding the role of regulatory element evolution in shaping the variation of gene regulation across species.

## Results

### Overview of the probabilistic framework for the birth-death evolutionary model of TFBS

Our goal is to detect lineage-specific rates of TFBS evolution and the branch of origin for individual TFBS. Here, lineage means any ancestral branch in the phylogeny or a branch leading toward any modern species. Our approach is to model TFBS evolution using a birth-death framework, in which individual TFBSs can be gained, lost, or conserved within a given lineage during evolution. The rate of TFBS creation (birth rate) and loss (death rate) are first estimated from a set of orthologous sequences, and are subsequently used to trace the evolutionary origin of individual TFBSs at the sequence level. The birth rate (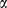) for a given motif represents the probability at which a TFBS appears at a single unoccupied site in a given year of evolutionary time. Similarly, the death rate (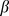) represents the rate at which an existing TFBS is lost per year. The method considers only TF motif counts within orthologous sequences across species, and therefore does not require an accurate base-to-base multiple sequence alignment. This framework allows us to reconstruct the ancestral states for each TFBS throughout the genome, providing a distribution for the branch of origin of the binding sites genome-wide.

For any set of orthologous sequences across species and a known phylogeny, we first estimate birth and death rates according to the observed numbers of TF motif occurrences within each species. Such orthologous sequences can, for instance, be obtained using a genome-wide multiple species alignment. However, the underlying base-level alignment is ignored once the orthologous sequences are obtained, and subsequently the model considers only the number of TF motifs within each sequence. Thus, the method operates independently of any details within the alignment once the sequence correspondence between species (i.e., orthologs) is obtained. Every node in the phylogeny is then associated with a (random) variable 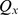, which represents the number of occurrences of the TFBSs at that node 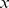. The value of 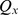 is known for each leaf node in the tree for any given ortholog set. Birth and death rates of a given motif can then be estimated by maximizing the likelihood across the entire data set, taking into account both branch lengths as well as the size of the sequence region (see Methods). Evolutionary rates were estimated using an iterative approach, but were found to be extremely robust according to the initial parameter settings. Once the birth and death rates are estimated using the full data set, we can use these rates to trace the branch of origin of individual TFBSs. This can be done by reconstructing the most likely ancestral state at each node of the phylogeny; i.e., the value of 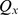 that maximizes the likelihood of the data for each individual ChIP-seq peak region. This provides the most likely number of TF motif occurrences at each node, and allows us to trace the most likely branch of origin for individual site. The overall procedure of our method works as follows. (1) We identify motif occurrences within ChIP-seq peak regions in human for a given TF. (2) We estimate the likelihood for each ancestral node in the phylogeny given the motif occurrences in the descendant species. (3) We determine the branch of origin for the TF-bound motif within ChIP-seq peak regions. See Methods and Supplementary Methods in Text S1 for details.

**Figure 1:**
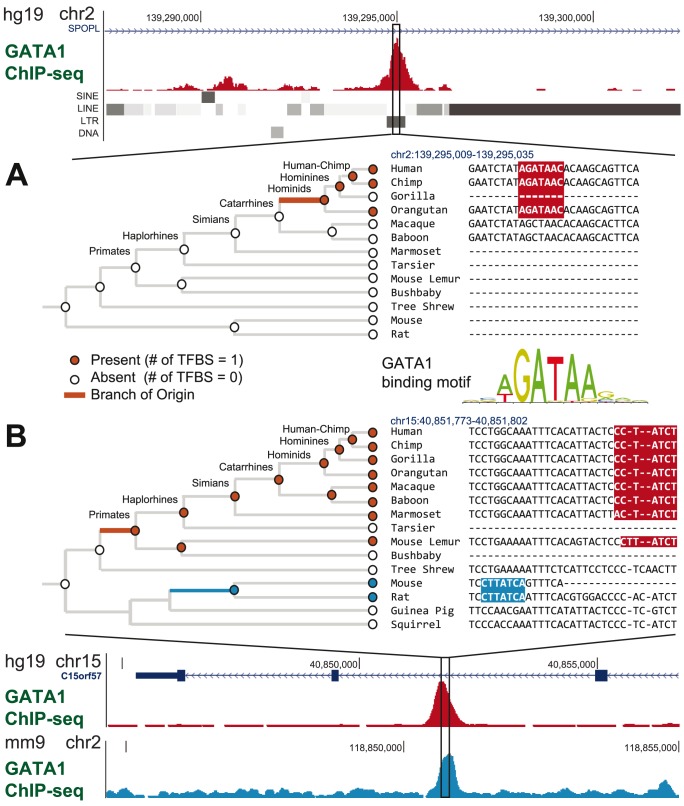
Model framework for lineage-specific GATA1 binding sites. Multiple alignments are shown for two GATA1-bound regions in humans. Red and blue boxes in the alignment correspond to GATA1 binding sites. Phylogenies illustrate the birth-death model framework, where the most likely number of binding sites is assigned to each ancestral node (denoted here as either ‘present’ or ‘absent’, at values 1 or 0 in this example). Highlighted branches denote the branch of origin. Evolutionary comparisons were conducted across ten primate species, as well as 36 non-primate vertebrates (not all are shown). (A) A binding site originating within an LTR insertion. (B) A genomic region containing a human GATA1 binding site originating along the ancestral primate lineage and a GATA1 binding site specific to mouse and rat. Despite nearly identical locations of the ChIP-seq peaks across human and mouse (in analogous Erythroblast cell lines), the ability of the method to identify specific branches of origin allows us to identify cases of TFBS turnover in close proximity.

The model framework and its motivations are illustrated in Figure 1. Figure 1A shows one scenario where the binding site was introduced to the genome through transposable elements (TEs) insertion followed by point mutation, which is most likely branch of origin of this site under our model. Figure 1B shows an example that our method is able to identify cases of TFBS turnover within stationary modules that might not otherwise be detected using human-mouse ChIP-seq data direct comparisons. In this genomic region, there is a human GATA1 binding site originating on the ancestral primate lineage and a GATA1 binding site specific to mouse and rat. Although the ChIP-seq peaks appear in the same location between human and mouse, our model can predict such lineage-specific events (which is also reflected in the cross-species alignment). Again, our algorithm predicted these branches of origin accurately without detailed alignment.

We note that in addition to the robustness of the parameter estimates of 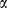 and 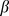, the branch of origin estimates were quite robust, since they were far more dependent on the number of sites in each species in each region (usually either 0 or 1) than the exact values of 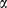 and 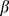. Although the loss of positional information would appear to make the approach insensitive to some cases of TFBS turnover, in which the creation of a new binding site coincides with the loss of an old binding site, in practice such cases are only problematic when applying the method to long branches in the phylogeny. For densely sampled phylogenies containing relatively short branch lengths, such turnover events can be inferred as long as the gain and loss occur on different branches. This is usually the case, since old binding sites are seldom lost through selection and are generally lost slowly, following a nearly-neutral rate of decay [29], [30]. Many neutrally (or near-neutrally) evolving sites are still present after relatively long periods of mammalian evolution [29].

### Large number of TFBS embedded in ChIP-seq peaks of a particular TF exhibit increased evolutionary rates along lineages leading to human

In this work, we applied our method to ChIP-seq data, which is now commonly used to map *in vivo* TF occupancy genome-wide [31]. We applied our method to ChIP-seq data sets for six TFs, namely GATA1, SOX2, MYC, MAX, ETS1, and CTCF [32]–[35]. These TFs were chosen, in part, for their diverse functional attributes, their well-documented binding motifs, and the availability of ChIP-seq data in analogous cell types in human and mouse. Using our method, we can determine cases in which there are lineage-specific differences in evolutionary rates of a given motif along a particular branch in the phylogeny. Since previous comparisons of ChIP-seq data from human and mouse have reported substantial divergence in protein-binding locations across the two species [5], [6], ChIP-seq peaks in human are likely to contain a high enrichment of TFBSs compared to the orthologous regions in more distantly-related species. We thus hypothesized that functional motifs present among ChIP-seq peak regions might be detectable by testing for an increased birth rate 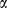 along lineages ancestral to humans relative to other lineages, since any recently-acquired TFBSs in humans would naturally increase the birth rate along these lineages.

To determine differences in the rate of motif evolution along specific lineages, we first assume a simple (null) model in which the birth and death rates (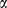 and 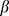) remain constant across the entire phylogeny. We can then compare this hypothesis to a model in which birth and death rates differ along a single branch of the phylogeny relative to the other branches. The statistical significance of lineage-specific evolutionary rates can then be assessed using a likelihood-ratio test [36], producing a P-value reflecting the significance of lineage-specific differences in evolutionary rates along that branch (Supplementary Methods in Text S1).

We applied this approach to human ChIP-seq data generated for the six TFs, testing for increased birth rates within the (−100,+100) region relative to the summit of the peaks. Orthologous regions were then determined using 46-way multiz alignments from the UCSC Genome Browser [37], and analyses were conducted using data from all 46 vertebrate lineages according to their known phylogeny. For every TF, with the exception of MYC, the known binding motif of TF was predicted with a substantially increased birth rate along branches ancestral to humans (P<1e-15). We note that in contrast to motif prediction using conservation-based approaches, our method generates motif predictions specifically using lineage-specific binding sites (or rather, their increased rate of creation along a specific lineage). For five of the six factors (GATA1, SOX2, MAX, CTCF, and ETS1), the documented binding motif of the TF produced the most statistically significant motif prediction using our method. The MYC binding motif, which has previously been noted for its strong patterns of conservation [27], was the only factor whose binding motif was not the top-ranked prediction, although it was still predicted under the P<1e-15 threshold. For each factor, we used an iterative method to generate a Position Weight Matrix (PWM) according to the nucleotide composition at each site of the motif within the (−100,+100) window of peaks in humans. These predicted PWMs very well matched with the known binding motifs as well as the results from the MEME suite [38] (Supplementary Methods in Text S1 and Table S1).

### Substantial number of human TFBSs have recent origins after the human-mouse divergence

**Figure 2:**
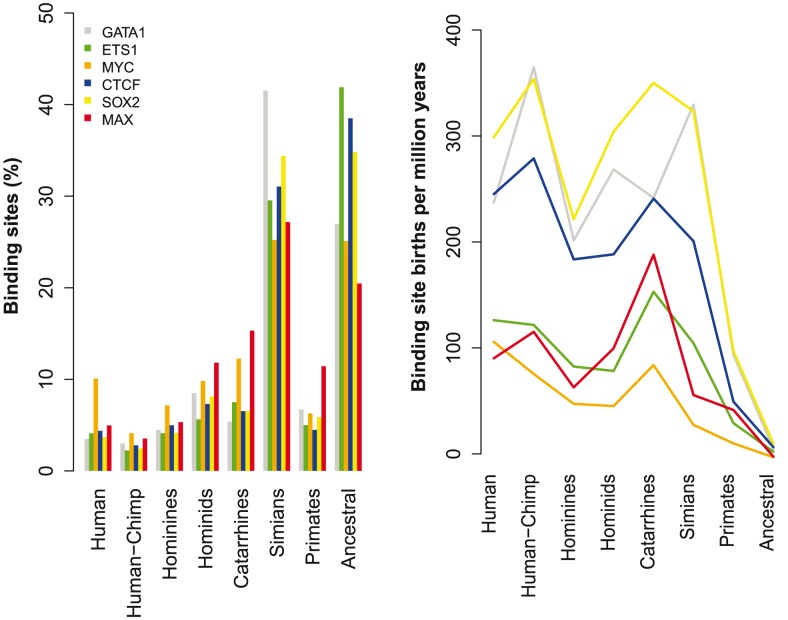
Time of origins for binding sites of six TFs in humans. Binding motifs were determined using human ChIP-seq data for GATA1, SOX2, MYC, CTCF, ETS1, and MAX. The branch of origin was determined for each binding site within the (−100,+100) region relative to a human ChIP-seq peak summit. (Left) Distribution of the branch of origin for each binding site. Branch labels correspond to those in Figure 1B. ‘Ancestral’ binding sites have origins prior to human-mouse divergence. (Right) The rate of binding site creation along branches ancestral to humans. Rates were estimated by dividing the number of sites originating along each branch by evolutionary time, including only binding sites currently existing in humans.

Using our approach, we sought to determine the branch of origin for each human binding site for the six TFs. Each binding site was thus either inferred to be present in the common human-mouse ancestor, or a more recent lineage leading to human using the phylogeny shown in Figure 1. The distribution of the branch of origin for each TFBS is shown in Figure 2. Notably, between ∼58–79% of all human TFBSs had inferred origins after the human-mouse split.

In addition to estimating the fraction of ancestral binding sites present in the human-mouse common ancestor, our approach allows us to estimate the age distribution across all binding site occurrences. As shown in Figure 2 (left panel), a sizeable fraction of binding sites in humans were estimated to have recent origins in primates. For instance, the fraction of human binding sites that are unique to hominids ranged from 16.1% for ETS1 to 31.1% for MYC. Additionally, the number of sites estimated to be human-specific, i.e., with recent origins after the human-chimp divergence, ranged from 3.5% to 10%.

Since the total number of protein-bound sites for each factor is quite large, these fractions represent a considerable number of sites unique to humans or closely-related primate lineages. The number of human binding sites originating since the common hominid ancestor ranged from 1,084 to 3,931 for each factor, including 440 to 1,409 human-specific binding sites. The rate of appearance for new sites along different ancestral lineages showed a relatively stable rate of creation for new binding sites for most TFs up through the common Simian-primate ancestor (Figure 2, right panel), with slight increase in more recent branches. The birth rate of new sites ranges from 50–300 per million years for most TFs.

Recent works have emphasized the emergence of new regulatory elements via transposable elements (TEs) [7], [39]–[41]. We assessed the amount of overlap between newly-derived TFBSs and annotated TEs determined by RepeatMasker [42]. Consistent with previous reports [7], [43], a substantial fraction of binding sites with recent origin were located within TEs. The number of TE-derived sites varied between factors, ranging from approximately 35–40% for recently-derived SOX2 and GATA1 binding sites to approximately 15–20% for newly-derived ETS1 and MYC binding sites. TE-derived TFBSs of specific branch of origins were associated with different TE families with different times of origin (Supplementary Results in Text S1 and Figure S1).

### TFBS origin estimates correspond to human-mouse ChIP-seq data and cross-species alignments

To assess the accuracy of the age estimates, we first compared our results to ChIP-seq data from human and mouse. Using analogous cell types across species, we determined the amount of overlap between human ChIP-seq peaks and ChIP-seq peaks in the orthologous regions in mouse. A human ChIP-seq peak was considered to be ‘shared’ with mouse if its summit was within 200 bp of a mouse ChIP-seq peak summit in the orthologous region (note that the mouse ChIP-seq data were not the input our algorithm). The amount of overlap was assessed separately for regions containing a human binding site present in the common human-mouse ancestor and for regions that are not ancestral.

We emphasize that, as illustrated previously in Figure 1B, ChIP-seq peaks shared across human and mouse can often contain TFBSs that are genuinely lineage-specific, since ChIP-seq peaks span a relatively broad region and can contain instances of TFBS turnover within static modules. In addition, human-specific ChIP-seq peaks can also contain ancestral binding sites, since such sites can either be lost (non-conserved) along the mouse lineage or may not be bound by the TF along that lineage.

Table 1 shows the amount of overlap in ChIP-seq peaks between human and mouse according to the estimated branch of origin of the TFBSs. Human peaks containing predicted ancestral TFBSs were far more likely to overlap with bound regions in mouse than peaks containing only predicted lineage-specific sites. Between 24–41% of human peaks that overlapped with a peak for the same TF in mouse contained only predicted lineage-specific TFBSs, while 59–76% of shared peaks contained a predicted ancestral TFBS. Thus, there was a clear enrichment for TFBSs predicted to be ancestral among the ChIP-seq peaks shared between human and mouse. Among human-specific ChIP-seq peaks, a substantially greater number contained only lineage-specific TFBSs than sites predicted to be ancestral to human and mouse.

**Table 1:** Human-mouse ChIP-seq factor-bound region overlap.

		Shared peaks (human-mouse)^b^				Lineage-specific peaks^c^			
Factor/Motif	Category^a^	Ancestral Sites^d^		Lineage-specific Sites^e^		Ancestral Sites^d^		Lineage-specific Sites^e^	
**GATA1**	**Human (Total)**	**433**	**(73.3%)**	**158**	**(26.7%)**	**6921**	**(34.9%)**	**12914**	**(65.1%)**
AGATAAG	*With Mouse*	*88*	*(14.9%)*	*24*	*(4.1%)*	*985*	*(5.0%)*	*623*	*(3.1%)*
	*TFBS-pair aligned*	*34*	*(5.8%)*	*11*	*(1.9%)*	*358*	*(1.8%)*	*93*	*(0.5%)*
**SOX2**	**Human (Total)**	**340**	**(75.7%)**	**109**	**(24.3%)**	**9451**	**(47.4%)**	**10487**	**(52.6%)**
WTAACAA	*With Mouse*	*123*	*(27.4%)*	*26*	*(5.8%)*	*2242*	*(11.2%)*	*1009*	*(5.1%)*
	*TFBS-pair aligned*	*85*	*(18.9%)*	*7*	*(1.6%)*	*747*	*(3.8%)*	*138*	*(0.7%)*
**MYC**	**Human (Total)**	**406**	**(58.5%)**	**288**	**(41.5%)**	**704**	**(26.4%)**	**1966**	**(73.6%)**
KCACGTG	*With Mouse*	*111*	*(16.0%)*	*39*	*(5.6%)*	*109*	*(4.1%)*	*93*	*(3.5%)*
	*TFBS-pair aligned*	*64*	*(9,2%)*	*16*	*(2.3%)*	*55*	*(2.1%)*	*38*	*(1.4%)*
**MAX**	**Human (Total)**	**420**	**(69.8%)**	**182**	**(30.2%)**	**1167**	**(21.2%)**	**4350**	**(78.9%)**
KCACGTG	*With Mouse*	*134*	*(22.3%)*	*49*	*(8.1%)*	*209*	*(3.8%)*	*269*	*(4.9%)*
	*TFBS-pair aligned*	*79*	*(13.1%)*	*19*	*(3.2%)*	*125*	*(2.3%)*	*66*	*(1.2%))*
**ETS1**	**Human (Total)**	**644**	**(73.5%)**	**232**	**(26.5%)**	**3885**	**(48.1%)**	**4189**	**(51.9%)**
MGGAAGT	*With Mouse*	*172*	*(19.6%)*	*35*	*(4.0%)*	*705*	*(8.7%)*	*283*	*(3.5%)*
	*TFBS-pair aligned*	*81*	*(9.3%)*	*9*	*(1.0%)*	*283*	*(3.5%)*	*58*	*(0.7%)*
**CTCF**	**Human (Total)**	**2008**	**(68.0%)**	**947**	**(32.0%)**	**3829**	**(41.2%)**	**5458**	**(58.8%)**
GGGGCKC	*With Mouse*	*766*	*(25.9%)*	*277*	*(9.4%)*	*770*	*(8.3%)*	*323*	*(3.5%)*
	*TFBS-pair aligned*	*256*	*(8.7%)*	*73*	*(2.5%)*	*231*	*(2.5%)*	*42*	*(0.5%)*

a The first row in each section gives the total number of ChIP-seq peaks with binding sites in humans within the (−100,+100) window separated into categories. The second row shows the number of these peaks also containing a TFBS in the orthologous regions in mouse, while the third row gives the number of aligned binding sites across the two species. Percentages are given with respect to the total number of shared and lineage-specific ChIP-seq peaks for each factor.

b Shared peaks are human ChIP-seq peaks within 200 bp of a ChIP-seq peak summit in the orthologous region in mouse. Analogous cell types were used across species (GATA1: Erythroblasts, SOX2: Embryonic stem cells, MYC, MAX, ETS1, CTCF: B-lymphocytes).

c Lineage-specific peaks in human are not within 200 bp of a mouse ChIP-seq peak in the analogous cell type. Only human peaks with identifiable orthologous regions in mouse were included.

d The numbers (and fractions) of human ChIP-seq peaks in each category containing binding motif occurrences estimated to be present in the human-mouse ancestor (‘Ancestral sites’).

e The numbers (and fractions) of human ChIP-seq peaks in each category containing only binding motif occurrences originating after human-mouse divergence (‘Lineage-specific sites’).

Although a relatively sizeable portion of shared ChIP-seq peaks contained only TFBSs predicted to be lineage-specific, in the majority of cases (>90%) the mouse TFBS did not occur within in sequence region orthologous to the human peak region used, but was instead offset to a non-overlapping region within a mouse peak. Very few of these TFBSs actually aligned across the two species, compared with those with predicted ancestral origin.

We compared the sequence level conservation of predicted TFBSs according to their branch of origins. We used the PhyloP mammalian conservation scores [44] available at the UCSC Genome Browser to determine the conservation level for TFBS in human. For a specific TF, we first computed the average PhyloP score (*X*) in each ChIP-seq peak and then calculated the average score (*M*) as well as standard deviation (*SD*) across all peaks in the genome. We then grouped the binding sites according to their branch of origin (in four groups: Hominid-specific, Simian-specific, Primate-specific, and Eutherian-specific) and calculated the average PhyloP score (*X*). Finally, we calculated the Z-score, i.e. (*X*-*M*)/*SD*). As expected, older binding regions show higher sequence level conservation than younger ones (Figure S2). These results suggest that our method can identify more recent, less-conserved TFBS, without relying on sequence-level conservation. Additionally, to further demonstrate the effectiveness of our method in identifying conserved TFBS, we directly compared with methods that use phylogenetic footprinting approaches. We compared with phylogenetic footprinting methods at both element level (using MotifMap [45] which is based on the method used in [46]–[48]) and module level (using PReMod [12]). Overall, our method outperformed both MotifMap and PReMod (see Supplementary Results in Text S1, Table S3, and Figure S5).

### Within-species variation is higher among TFBSs of more recent origin

**Figure 3:**
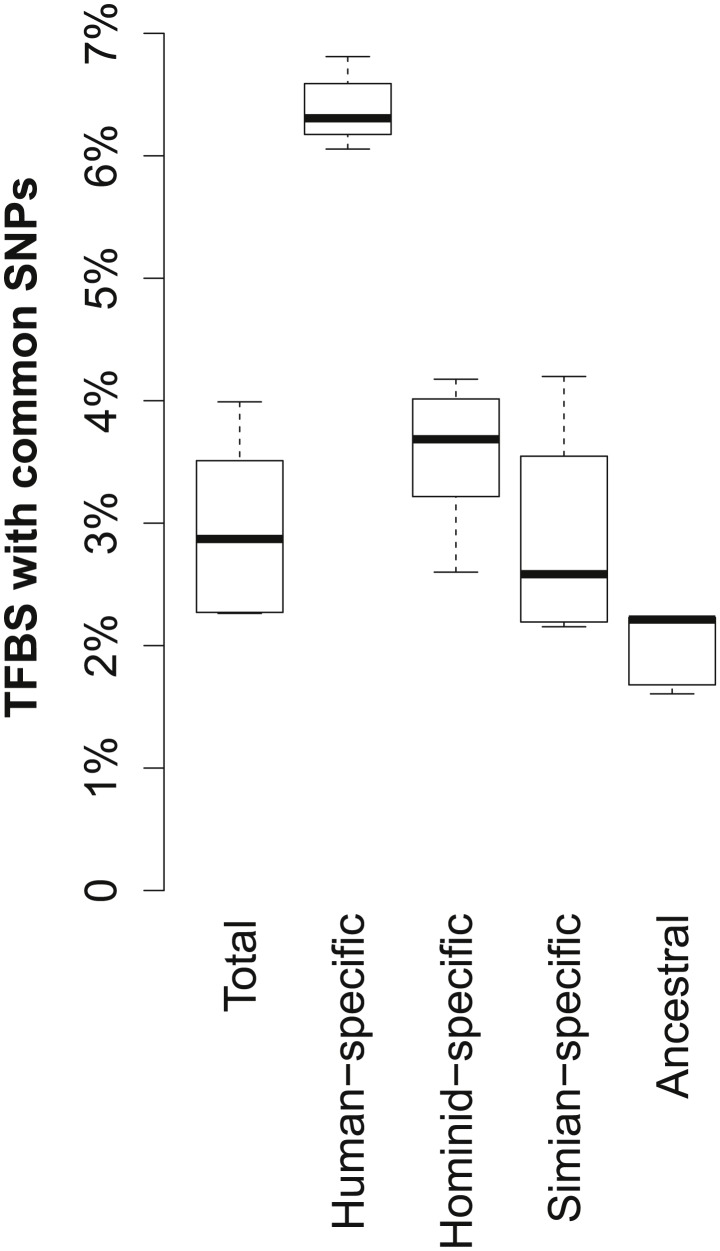
Within-species variation of binding sites according to time of origin. Boxplots show the fraction of TFBSs containing common SNPs in humans [72], where plots show the median (center line), upper- and lower-quartile (boxes), and range (whisker extremes) of percentages across the TFBSs of six TFs. TFBSs are categorized as human-specific, hominid-specific (not including human-specific sites), Simian primate-specific (not including hominid-specific sites), and ancestral (present in the human-mouse common ancestor). Overall fractions (including all sites) are shown in the left-most boxplot. Note the substantial rise in the amount of human variation within more recently derived binding sites compared to older sites.

Recent work has reported a substantial difference between genomic locations that are conserved across species versus those conserved within the human population [26]. Thus, we compared both human variation data as well as sequence conservation across primates to the relative age of the TFBSs. Comparing the overall frequency of common SNPs in humans among TFBSs originating at different times of evolution showed that a substantial fraction of human-specific TFBSs contained common SNPs, comprising over 6% of all human-specific TFBSs (Figure 3). This is much higher than the total fraction of TFBSs overlapping with a common SNP, at a median of less than 3% across all six factors.

Since substantial variation exists in TF-binding events between human individuals [9], this high amount of variation among human-specific binding sites may partially reflect the fact that some TFBSs inferred to be human-specific may not be shared by the entire human population. However, recently-derived TFBSs in hominids were also substantially enriched for common SNPs, even when excluding human-specific TFBSs. For instance, among hominid-specific binding sites that are not human-specific, with a median of almost 4% of all sites. As these sites are shared across species, they cannot be fully explained by variation within the population. In contrast, common SNPs were consistently low among TFBSs with origins prior to hominids (Figure 3). Note that this observation was not biased by the SNP density surrounding the binding sites (Figure S3).

### Hominid-specific binding sites target specific biological processes

To determine potential functions for the newly derived binding sites, we tested whether genes predicted to be targeted by binding sites with recent origins in hominids were involved in specific biological processes or pathways. Such enrichment was determined for genes near hominid-specific binding sites compared to the total list of protein-bound sites for each factor, where each TFBS was mapped to the nearest TSS, up to a distance of 100 kb. This allowed us to assess potential lineage-specific functions of these sites relative to sites of more ancient origin.

Genes located nearest to hominid-specific binding sites were more frequently enriched for neural and sensory-related functions, and were in many cases involved in neurological pathways (Table S2). CTCF, MYC, and SOX2 target gene sets were all enriched for GO categories involved in sensory perception, while GATA1, MYC, ETS1, and MAX were enriched for neural development and differentiation categories. Among the six factors, neural-related functions are only well-documented for SOX2, which is involved in neuronal-cell maintenance [49], [50] and whose hominid-specific target sites are enriched genes involved in sensory perception. Similarly, genes in proximity to hominid-specific binding sites for CTCF and MYC were enriched for sensory perception processes and pathways, particularly those related to olfaction, and in the case for MYC, hominid-specific target genes were also enriched for genes involved in synapse assembly and receptor clustering and binding. Hominid-specific binding sites for GATA1, most commonly known for its role in erythroid differentiation [51], were also found enriched near genes involved in axon extension of neural cells. For ETS1, hominid-specific binding sites were near genes involved in spinal cord neuron differentiation, ventral spinal cord development, and behavioral fear response. We also found that the hominid-specific sites are near genes in different pathways as compared to Simian-specific sites and more ancestral sites (Table S4 and Table S5).

### Branches of origin of CTCF binding sites differ between cell type specific and ubiquitously bound sites

**Figure 4:**
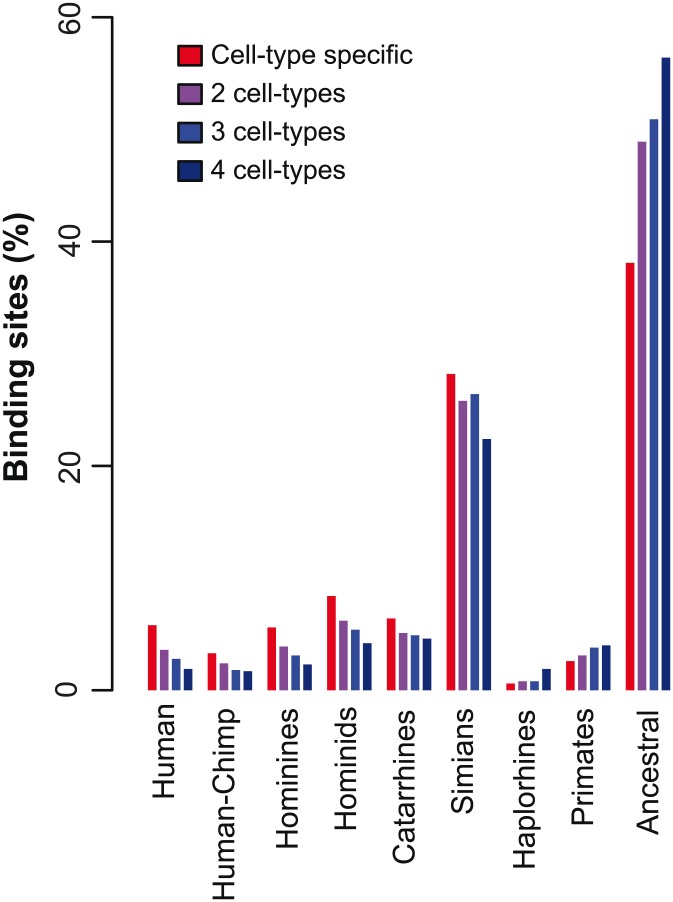
Time of origin for human CTCF binding sites according to cell-specificity. CTCF binding sites in humans were separated according to cell-specificity, considering four distinct cell lines (GM12878, H1hESC, HAC, and HRE). Colored bars correspond to varying amounts of cell-specificity, denoting sites bound in one, two, three, or in all four cell types (red to dark blue bars, respectively). Note the tendency for cell-specific binding sites to have more recent evolutionary origins than sites bound ubiquitously in all cell types.

ChIP-seq data of CTCF is available for several different cell types, and thus we sought to determine whether CTCF-bound sites have distinct age distributions according to cell type. We thus expanded our analyses to include ChIP-seq data for CTCF collected from four different cell types in humans: B-lymphocytes (GM12878), embryonic stem cells (H1hESC), cerebellum (HAC), and kidney cells (HRE). Interestingly, we observed substantial differences in the age distribution of cell-type specific and ubiquitously-bound sites. Figure 4 shows the age distribution for CTCF-bound sites, separated according to the number of cell types in which each site was bound. Only 43% of all sites bound by CTCF in all four cell types were primate-specific and 10% were specific to hominids. For cell-specific sites, i.e., those bound by CTCF in only one cell-type, these fractions increased to 63% and 24%, respectively (Figure 4).

These results are consistent with previous observations that cell-type specific CTCF binding sites are less conserved across mammalian species than ubiquitously-bound sites [39], [52]. However, the recent origin of the cell-type specific TFBSs suggests that this cannot simply be explained by a relative lack of selective pressure, since cell-specific CTCF binding sites were frequently found to be absent in older lineages. Instead, there exists the possibility that cell type specific TFBSs might contribute to lineage-specific regulatory function.

### Our method identified a TFBS turnover event within a functionally conserved enhancer

**Figure 5:**
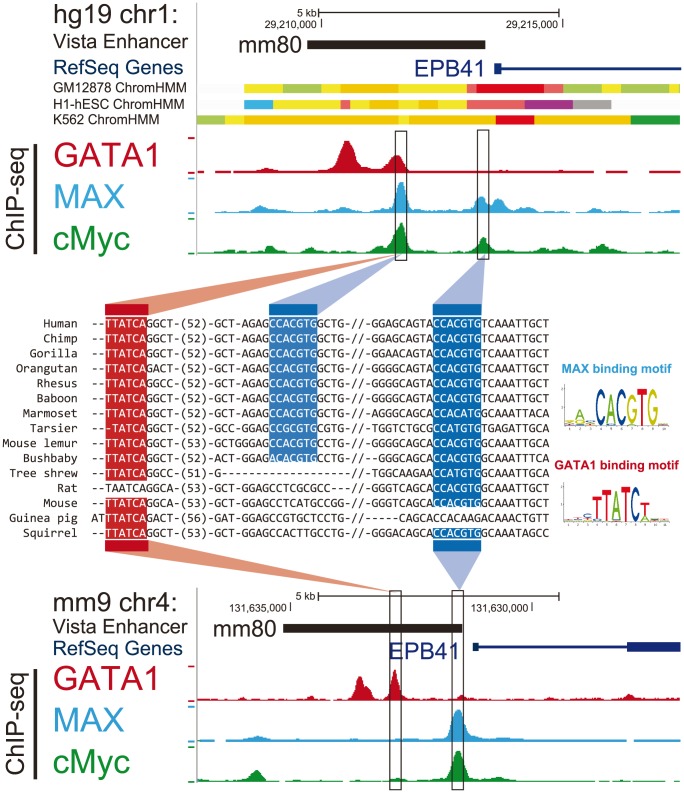
A TFBS turnover event within a functionally conserved enhancer. A TFBS turnover event shows the impact of lineage-specific TFBS within an enhancer. The Genome Browser view shows the upstream of human gene EPB41. VISTA Enhancer track and ChromHMM track (orange means strong enhancer, yellow means weak enhancer) indicate a putative human enhancer. ChIP-seq signals of three TFs used in this study near predicted enhancer region are consistent with predicted lineage-specific binding site represented by 46-way multiple sequence alignment (only a subset of species are shown). Note that here the two MAX binding sites are also MYC binding sites since MAX and MYC have very similar motif. A potential TFBS turnover is observed between two predicted MAX/MYC binding sites (1700 bp apart). Different TFBSs are highlighted in different colors with MAX in blue and GATA1 in red. The predicted enhancer may function as blood cell specific enhancer in mouse, demonstrated by images of LacZ positive E11.5 mouse transgenic embryo on the VISTA Enhancer Browser [54] (ID: mm80; http://enhancer.lbl.gov/cgi-bin/imagedb3.pl?form=presentation&show=1&experiment_id=80&organism_id=2).

Using our framework, we then utilized genome-wide chromatin data to search for potential functional consequences driven by birth or death of specific lineage-specific TFBS. We intersected the lineage-specific TFBSs with predicted human enhancer regions marked by ChromHMM model [53] as well as *in vivo* verified enhancers listed in the VISTA Enhancer Browser [54]. Figure 5 shows a potential functional take-over through TFBS turnover inside an enhancer after human-mouse divergence. At the sequence level, two MAX binding sites were identified by our method with an ancestral one and a primate-specific binding site emerging after human-bushbaby split (Figure 5). Here these two MAX binding sites are also MYC binding sites since MAX and MYC have very similar motif (their ChIP-seq peaks overlap in Figure 5). The orthologous region of predicted primate-specific MAX/MYC binding site has no MAX or MYC ChIP-seq signal at all in mouse, which is consistent with our lineage-specific prediction. Since the young MAX/MYC binding site only locates 1,700 bp upstream of the ancestral one and ChIP-seq intensity of ancestral binding site is much weaker in human compared to mouse, this is likely to be a turnover of MAX/MYC binding site within the enhancer. Then we asked whether the function of predicted enhancer was conserved between human and mouse. Interestingly, despite the potential turnover of MAX/MYC binding site in the sequence level, the mouse orthologous region of predicted enhancer was found to drive reproducible LacZ expression in E11.5 mouse blood cell as demonstrated by *in vivo* transgenic mouse embryos essay based on VISTA Enhancer Browser, which confirms that the predicted enhancer also functions as an enhancer in mouse. It certainly remains to be solved whether this enhancer regulates the same genes in human and mouse and why the ChIP-seq signal on ancient MAX/MYC binding site is much weaker than the younger one in human. Nevertheless, this example demonstrates the ability of our method to compare functional level dynamics with sequence level difference in an evolutionary framework.

## Discussion

The approach presented here represents an initial step towards understanding regulatory element evolution using *in silico* methods without relying on accurate cross-species alignments. Studies regarding the evolution of TFBSs are largely separable into those that emphasize cross-species conservation of regulatory elements [13], [27], [55]–[57] and studies highlighting the substantial divergence of transcription factor-binding events across species [5]–[7], [58]–[60]. To some extent, this dichotomy may largely reflect differences between analyses conducted *in silico* and experiment-based studies. Although computational approaches have had some success in identifying cis-regulatory alterations responsible for changes in phenotype [16], [17], the study of regulatory sequence evolution is limited by reliance upon multiple sequence alignments [20], [21]. Reconstructing the ancestral states of regulatory sequences is a particularly challenging problem; comparative studies of regulatory elements generally categorize sites as ‘conserved’ and ‘non-conserved’ in terms of their presence across species [34], [39], [61]. ‘Non-conserved’ sites are thus often assumed to be under a weaker amount of purifying selection, indicating a relative lack of function [62]. However, any interpretation of the results is obscured by the fact that no distinction can be made between ancestral sites that have later been lost, versus sites of recent origins. Newly-derived functional elements, which are also ‘non-conserved’ by the common definition, may induce a gain-of-function trait, harboring the potential for lineage-specific adaptation or positive selection. It has long been argued that alterations in regulatory function are responsible for many, if not most, species-specific traits [1]–[3], and it is indeed these elements that are likely to contribute to phenotypic adaptation and the variation seen across species.

In this context, the high fraction of TFBSs that have recent origins after human-mouse divergence is particularly notable. For all six factors analyzed, the majority of human TFBSs bound *in vivo* were originally absent in human-mouse common ancestor, which is consistent with previous cross-species comparisons noting substantial divergence in ChIP-seq protein-binding events across the two species [5], [6] and similar comparisons presented here (Table 1), and is also comparable to detailed analyses conducted in *Drosophila* using alternative approaches [57]. This does not appear to simply be a consequence of the specific selection of TFs, since binding motifs for several factors analyzed (CTCF, ETS1, MYC, MAX) have been previously documented as ‘highly conserved’ compared to motifs of other factors [27], [39]. In particular, a comprehensive scan for conserved motifs identified the binding motifs of MYC and ETS1 as the second and third-highest ranking motifs across all motifs in terms of conservation score across human, mouse, rat, and dog (where the ETS1 binding motif was denoted as the ELK1 motif) [27]. It is important to note that ‘phylogenetic footprinting’ approaches usually measure motif conservation relative to neutrally evolving elements in non-coding regions. The fact that such motifs are substantially more conserved compared to a neutral proxy does not mean that the majority of binding sites are conserved, nor does it imply that protein-bound sites considered collectively across the genome are more conserved than coding sequences under relatively weak selection. Since some of the most ‘highly conserved’ regulatory motifs are largely comprised by sites with recent origins, it is unlikely that this birth of new binding sites is simply a special property of a handful of TFs, but instead is likely to apply across many other factors. Nevertheless, some analysis challenges remain. For example, it is acknowledged that not all computationally predicted TFBSs within ChIP-seq peaks were actively bound by the TF and many ChIP-seq peaks may correspond to experimental noise. While researchers have continuously improved TFBS identification, high-throughput experimental approaches would be necessary to systematically validate binding site predictions, especially for lineage-specific ones.

Among TFBSs in humans, a considerable amount of them are unique to hominids and are even human-specific. Since there are an estimated ∼1700–1900 TFs in the human genome [63], if even a small fraction of these sites harbor important regulatory potential, the total number of human-specific functional binding sites genome-wide is quite large. Although not all TFBSs with recent origins will be responsible for lineage-specific traits, these results nonetheless offer the potential to understand adaptive evolution of gene regulation via the creation of new TFBSs, not only alone, but also in combination. In addition, a number of recent studies have highlighted differences in transcription factor binding within humans [9], [64], and our results suggest that sequence-level variation of TFBSs within the population may be more common among more recently-derived binding sites.

The functional categories of genes close to recently derived binding sites may serve to shed new light upon adaptive traits obtained along the lineage leading to human. It is interesting that, despite substantial differences in the biological functions generally associated with the six TFs analyzed, genes near binding sites with recent origins are enriched for several sensory and neural related pathways and processes. We note that since the ChIP-seq data we used in this study were not derived from neuronal cells, further study is needed to more comprehensively understand the roles of hominid-specific sites in specific cell types. Nevertheless, identifying such lineage-specific regulatory elements not only provides potential insight on human biology, but may also provide new knowledge on the molecular mechanisms of human diseases.

A natural future direction for this work would be to determine the specific regulatory effects of the recently derived TFBSs identified using this method. For instance, enrichment for within-species variation among recently derived binding sites raises the intriguing possibility that recently derived TFBSs most responsible for phenotypic differences across species are also the elements responsible for within-species variation. Future work will be necessary to demonstrate whether this is the case and the differences in traits brought about by this variation. Also, our current model needs to be integrated with gene expression data to understand the interplay between cis-regulatory element evolution (e.g., binding site turnover and lineage-specific sites) and gene expression differences across different species [65]–[68]. The extent to which newly derived TFBSs operate primarily as cell-type specific elements with cell-specific regulatory function also remains an open question. The mechanisms that contribute to cell-type specific TF binding, whether through the presence or absence of other protein factors, accessibility of DNA within the chromatin structure, or by other means, are also possible future directions that can be more fully understood using a combination of different types of data that are becoming available.

## Methods

### The model

Our approach is designed to estimate genome-wide rates of evolution for a given motif according to a birth-death framework (formally, a quasi birth-death process [69]), similar to that used to measure the timing of accelerated motif evolution as in [29]. The birth rate (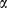) represents the rate at which a new motif occurrence appears at any unoccupied site per year, while the death rate (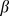) represents the rate at which an existing site is lost per year. Given a set of orthologous sequences and a known phylogeny, we estimate birth and death rates for the motif across the phylogenetic tree using a maximum likelihood approach.

Let 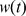 denote the probability that a given TFBS motif occupies that nucleotide position at time 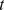
_._ The probability that the motif will exist at time 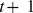 is then
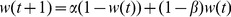
(1)Setting 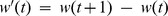 to be the rate of change of 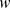 with respect to 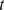, Eq (1) gives the differential equation
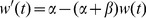
(2)We denote the solutions to Eq (2) by 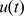 and 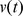, where 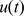 assumes that the motif was present at this site at time 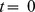, while 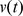 assumes that the motif did not exist at time 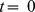 (i.e., initial conditions 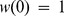 and 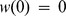, respectively). As 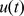 and 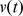 are solutions for 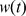 in Eq (2), both represent the probability that the motif exists at a specific nucleotide position after time 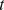, differing only in the initial conditions. Solving Eq (2) gives
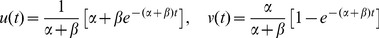
(3)


The transition probability 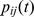 is the conditional probability that a given region will contain 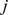 occurrences of the motif after time 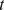, assuming 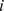 initial occurrences of the motif within the region. Namely, if a sequence initially contains 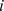 motif occurrences, the probability 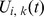 that 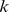 of these occurrences remain after time 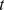 is given by the binomial distribution:
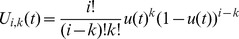
(4)Similarly, if the width of our region is 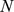 nucleotide sites, there are initially 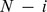 unoccupied sites. Thus the probability 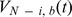 that 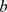 of these unoccupied sites become occupied after time 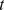 also follows the binomial distribution:
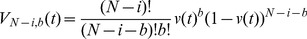
(5)


The transition probability 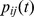 that the given region contains 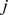 sites after time 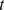 is then given by
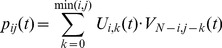
(6)Here, the sum is over all possible values 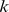, where 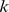 represents the number of motif occurrences at time 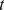 among the sites that were originally occupied at time 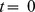.

### Calculating the likelihood of the data

Given the birth and death rates (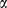 and 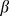) across the tree (which are estimated using the method described below), we can calculate the likelihood of the data using Felsenstein's pruning algorithm [70]. Let us first consider data from a single sequence. We let 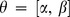 represent the parameter vector comprising the birth and death rates, and let 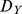 represent the data downstream of a node 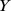 in the phylogeny. Let 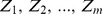 be the daughter nodes of 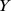, occurring at times 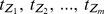 relative to parent node 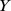, respectively.

If random variable 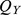 represents the number of motif occurrences at node 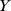, the likelihood 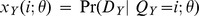 of the data downstream of 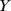
_,_ assuming 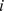 motif occurrences exist at node 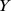
_,_ can be obtained recursively. This likelihood is given by
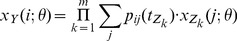
(7)where the inner sum is across all possible values for 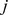, corresponding to the number of motif occurrences at daughter node 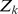. If node 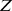 is a modern lineage, the probability 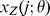 is equal to 1 if we actually observe 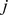 motif occurrences within the sequence, while the likelihood is zero otherwise.

The likelihood of the data can therefore be obtained recursively by determining the values 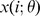 progressively for each node farther up the tree. The log-likelihood 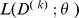 for a single sequence (the 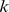
th sequence) is then given by
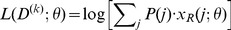
(8)where 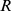 is the root node and 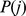 is the prior probability that 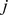 binding sites exist in a single sequence. For our implementation, prior probabilities 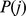 were set to the Poisson distribution: 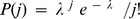 where 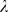 is the mean number of motif occurrences per sequence. The total log-likelihood 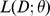 is then the sum 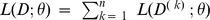 across each of the 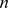 regions.

### Determining the optimal ancestral states

We can determine the most likely ancestral states using the computed values for 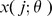 at each node in the phylogeny. At the root node 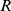, the most likely ancestral state is the one that produces the highest likelihood; that is, the value of 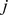 that maximizes the expression 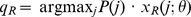. Progressively moving down the tree, if the most likely number of motif occurrences at parent node 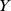 is 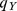, the optimal number of motif occurrences 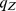 at a daughter node 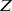 is given by
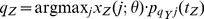
(9)where 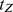 is the branch length from node 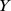 to node 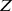.

### Birth-death rate estimation

Birth and death rates can be estimated using a maximum-likelihood approach. Namely, we use an EM-based approach [71] to iteratively optimize the likelihood of the data 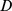 given the parameters 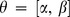. We begin with an initial estimate 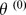 for the birth-death rates, generated by determining empirical birth-death rates after conducting ancestral reconstruction using parsimony (in our analysis, we found that the optimized parameters were not sensitive to the initial estimates). We determine the most likely ancestral state at each node given the initial parameter values. We then determine the observed number of births and deaths according to these optimal ancestral states, providing new estimates for the birth and death rates 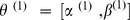. We then continue the process, using the previous parameter estimates 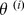 at each iteration to estimate the optimal ancestral states and obtain more optimal estimates of the birth and death rates 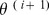 until convergence (i.e., where 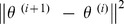 falls below a certain threshold).

## Supporting Information

Figure S1 **Fraction of binding sites overlapping transposable elements.** Plots show the percentage of human TFBSs overlapping transposable elements (TEs) (y-axis), where binding sites are separated according to the branch of origin (x-axis). (A) Each colored plot corresponds to a single consensus motif; the fraction of binding sites overlapping TEs documented by RepeatMasker [42] are given as percentages along the y-axis. (B) Genome-wide prevalence of seven common TE families in humans. Fractions denote the total number of sites derived from each family across all TEs documented by RepeatMasker. (C) TE family composition for TE-derived TFBS according to the age of origin of SOX2, GATA1, and CTCF binding sites. The total height of each plot shows the total fraction of TFBS overlapping known TEs. Colored regions corresponding to the colored regions of Panel B denote the fraction of TFBS derived from each TE family according to the estimated age of the binding sites (x-axis).
(TIF)

Figure S2 **PhyloP conservation vs. TFBS with different branch of origins.** We used the PhyloP mammalian conservation scores available at the UCSC Genome Browser to determine the sequence conservation level for TFBS with different branch of origins in human. X-axis shows TFBS with different branch of origins for four different window sizes surrounding the peak summit. Y-axis shows the Z-score distribution for each group. For a specific TF, we first computed the average PhyloP score (*X*) in each ChIP-seq peak and then calculated the average score (*M*) as well as standard deviation (*SD*) across all peaks in the genome. We then grouped the binding sites according to their branch of origin (in four groups: Hominid-specific, Simian-specific, Primate-specific, and Eutherian-specific) and calculated the average PhyloP score (*X*). Finally, we calculated the Z-score, i.e. (*X*-*M*)/*SD*. t-statistic from t-test between the youngest and the oldest for each group is also shown.
(TIF)

Figure S3 **Background SNP density for TFBSs with different branch of origin.** TFBSs were grouped into different branches of origin (X-axis). To calculate the background SNP density surrounding these TFBSs, we extended 1 kb, 500 bp, or 125 bp to both directions (i.e., 2k, 1k, or 250 bp window) and counted the number of common SNPs in this 2 kb window. The figure shows that there are no significant differences of SNP density surrounding the TFBSs with different branches of origin.
(TIF)

Figure S4 **Positive enhancer rate based on the VISTA enhancer database.** The positive rate is the percentage of human enhancers that also show enhancer activity in mouse. The expected rate is based on all the enhancers overlapping with TFBS used in our six TF data sets. ‘More ancestral enhancer’ has higher positive enhancer rate compared with ‘more lineage-specific enhancers’ or ‘neutral enhancer’, which is generally consistent with our computational prediction that the ancestral TFBS are more functionally conserved than lineage-specific TFBS, even though this comparison dataset is not ideal. See Supplementary Results in Text S1 for details.
(TIF)

Figure S5 **Comparison between our method and MotifMap.** A receiver operating characteristic (ROC) curve shows the prediction power between our method and MotifMap. ROC curves for MotifMap were generated using different BBLS thresholds (ranging from zero to the maximum possible BBLS score here, 4.73) to call a TFBS as a conserved one. In our method, we tested two shift sizes, +/−15 bp (light blue) and +/−30 bp (dark blue). The results from MotifMap were based on +/−15 bp shift size (magenta). See Supplementary Results in Text S1 for detailed explanation of the comparison method and how the benchmark dataset was constructed.
(TIF)

Figure S6 **Sensitivity of our method when we add noisy sites in leaf nodes.** Sensitivity of our method on the uncertainty of number of binding sites in leaf nodes was determined by randomly deleting/adding 5% sites in +/−100 bp of peak summit in all the species except human. Predictions were compared with original results and the changes of branch of origin for each binding site were counted. Y-axis shows the percentage of binding sites with various branch change. 0 means the prediction of branch of origins remain same before and after we randomly delete or insert binding sites.
(TIF)

Figure S7 **Sensitivity of our method when we change the branch lengths.** Sensitivity of our method on the branch length of phylogenetic tree was characterized by randomly changing the branch lengths to a certain extent. The length of each branch in phylogenetic tree can be varied in certain range relative to its original length (shown on X-axis). Y-axis shows the percentage of binding sites that have different branch of origin before and after we randomly change branch lengths in the phylogenetic tree.
(TIF)

Table S1 **Comparison of consensus motifs.**
(PDF)

Table S2 **Gene functions and pathways associated with hominid-specific TFBS.**
(PDF)

Table S3 **Performance comparison with MotifMap and PReMod.**
(PDF)

Table S4 **Gene functions and pathways associated with simian-specific TFBS.**
(PDF)

Table S5 **Gene functions and pathways associated with ancestral TFBS.**
(PDF)

Text S1 **Supplementary methods and supplementary results.**
(PDF)
