# Supplementary figures and images for: Tracing the Evolution of Lineage-Specific Transcription Factor Binding Sites in a Birth-Death Framework
Source: PLoS Comput Biol. 2014 Aug 21;10(8):e1003771. doi: 10.1371/journal.pcbi.1003771 (PMC4140645; doi:10.1371/journal.pcbi.1003771)

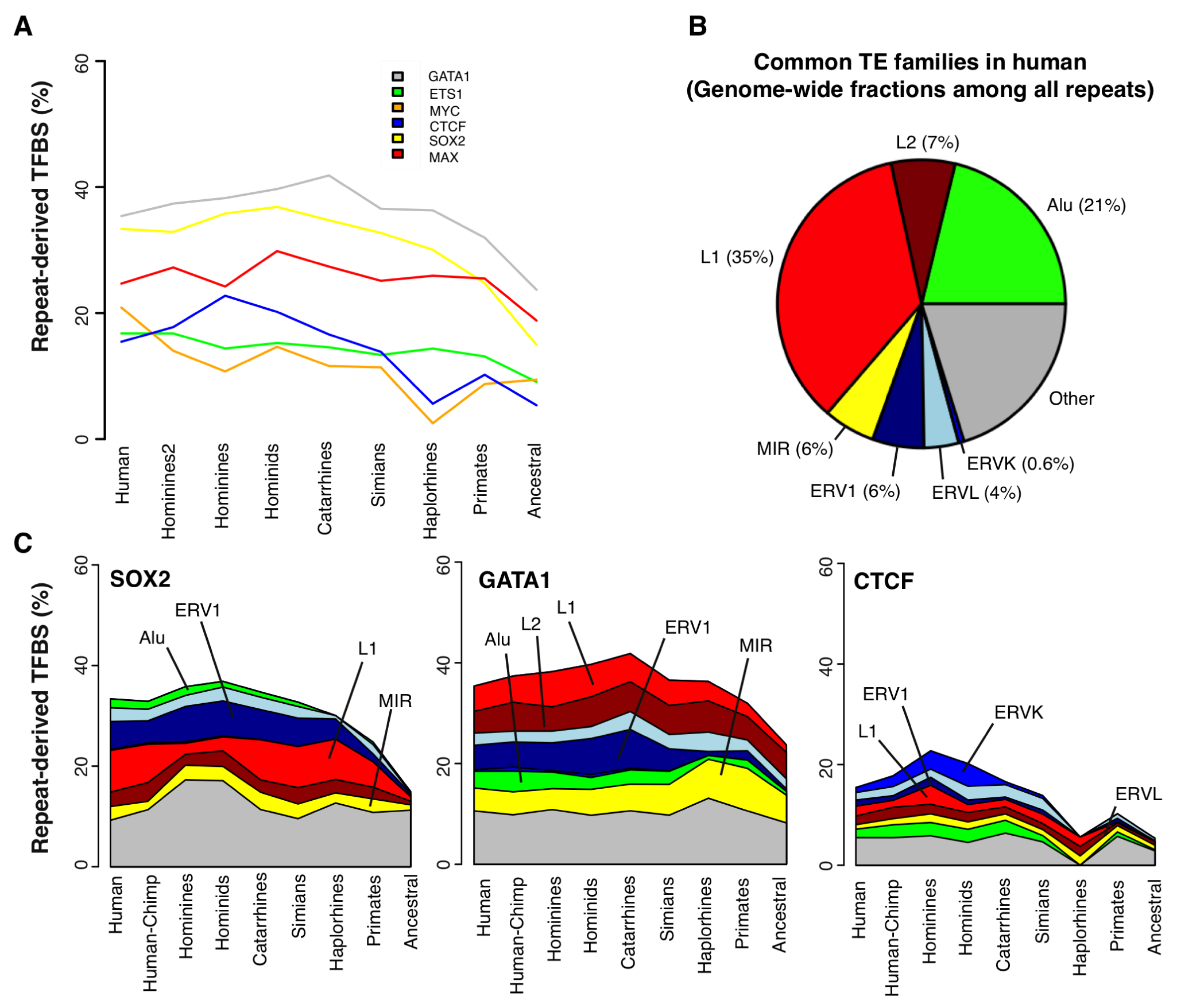

Supplement: Figure S1 — Fraction of binding sites overlapping transposable elements. Plots show the percentage of human TFBSs overlapping transposable elements (TEs) (y-axis), where binding sites are separated according to the branch of origin (x-axis). (A) Each colored plot corresponds to a single consensus motif; the fraction of binding sites overlapping TEs documented by RepeatMasker [42] are given as percentages along the y-axis. (B) Genome-wide prevalence of seven common TE families in humans. Fractions denote the total number of sites derived from each family across all TEs documented by RepeatMasker. (C) TE family composition for TE-derived TFBS according to the age of origin of SOX2, GATA1, and CTCF binding sites. The total height of each plot shows the total fraction of TFBS overlapping known TEs. Colored regions corresponding to the colored regions of Panel B denote the fraction of TFBS derived from each TE family according to the estimated age of the binding sites (x-axis). (TIF) [file pcbi.1003771.s001.tif]

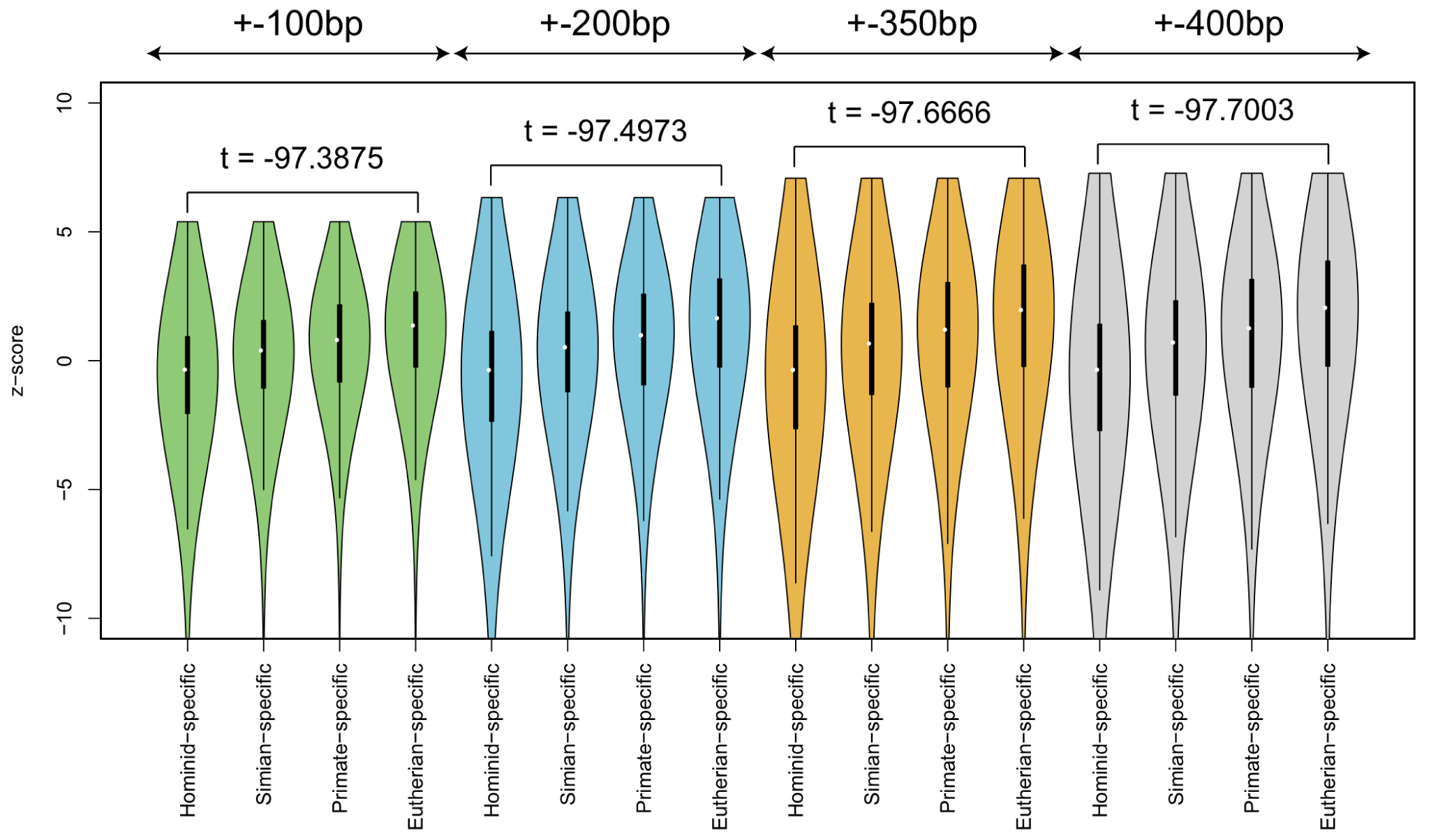

Supplement: Figure S2 — PhyloP conservation vs. TFBS with different branch of origins. We used the PhyloP mammalian conservation scores available at the UCSC Genome Browser to determine the sequence conservation level for TFBS with different branch of origins in human. X-axis shows TFBS with different branch of origins for four different window sizes surrounding the peak summit. Y-axis shows the Z-score distribution for each group. For a specific TF, we first computed the average PhyloP score (X) in each ChIP-seq peak and then calculated the average score (M) as well as standard deviation (SD) across all peaks in the genome. We then grouped the binding sites according to their branch of origin (in four groups: Hominid-specific, Simian-specific, Primate-specific, and Eutherian-specific) and calculated the average PhyloP score (X). Finally, we calculated the Z-score, i.e. (X-M)/SD. t-statistic from t-test between the youngest and the oldest for each group is also shown. (TIF) [file pcbi.1003771.s002.tif]

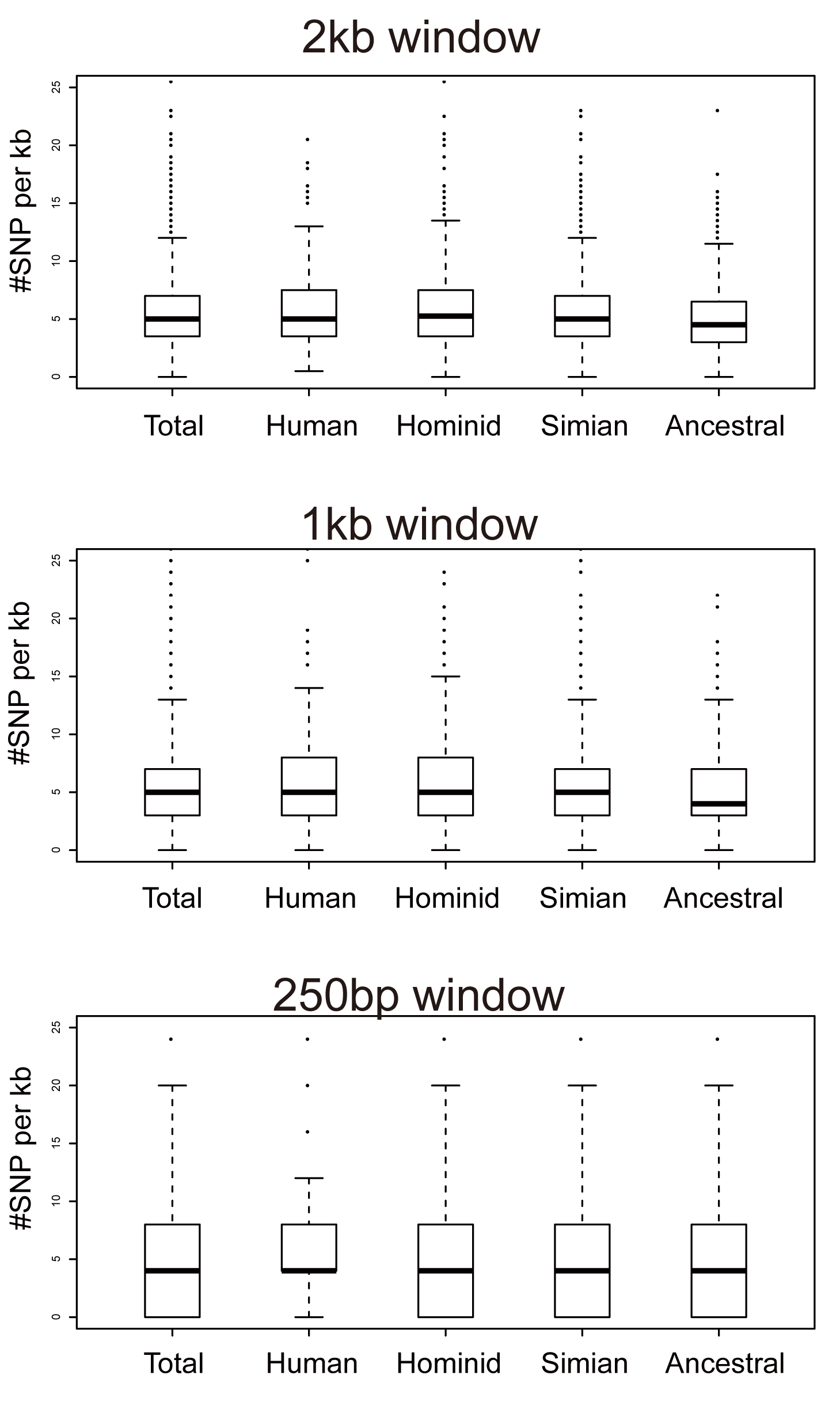

Supplement: Figure S3 — Background SNP density for TFBSs with different branch of origin. TFBSs were grouped into different branches of origin (X-axis). To calculate the background SNP density surrounding these TFBSs, we extended 1 kb, 500 bp, or 125 bp to both directions (i.e., 2k, 1k, or 250 bp window) and counted the number of common SNPs in this 2 kb window. The figure shows that there are no significant differences of SNP density surrounding the TFBSs with different branches of origin. (TIF) [file pcbi.1003771.s003.tif]

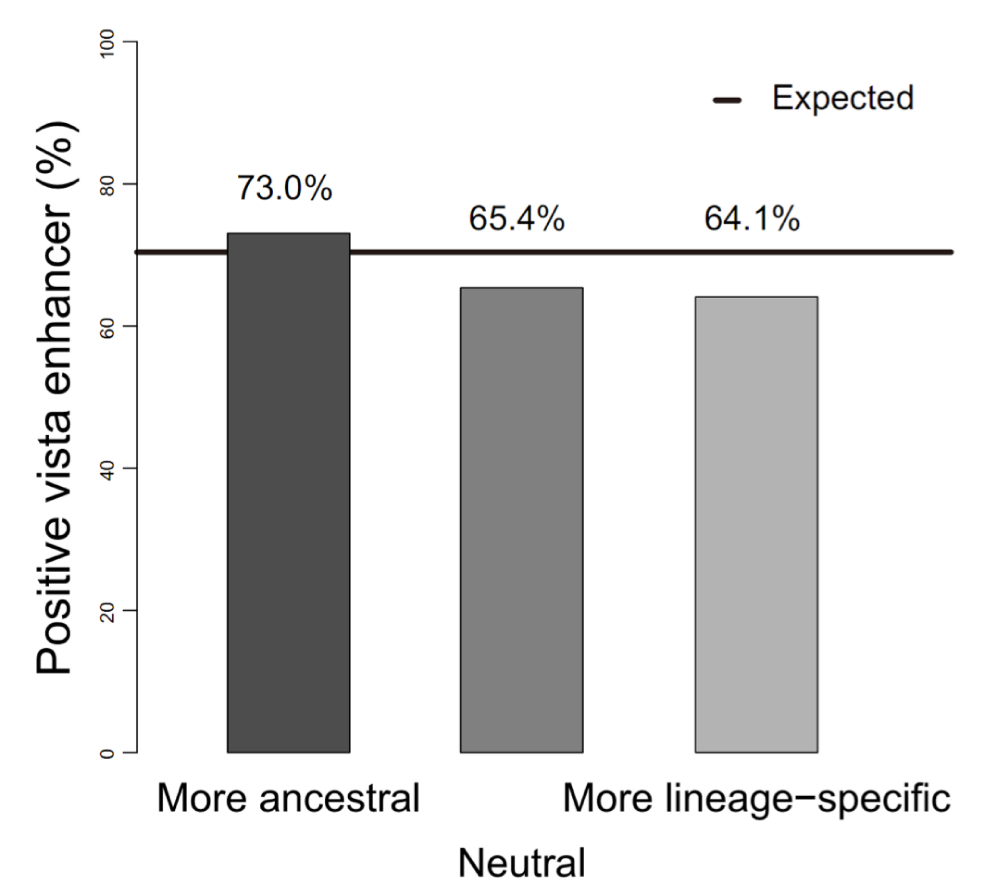

Supplement: Figure S4 — Positive enhancer rate based on the VISTA enhancer database. The positive rate is the percentage of human enhancers that also show enhancer activity in mouse. The expected rate is based on all the enhancers overlapping with TFBS used in our six TF data sets. ‘More ancestral enhancer’ has higher positive enhancer rate compared with ‘more lineage-specific enhancers’ or ‘neutral enhancer’, which is generally consistent with our computational prediction that the ancestral TFBS are more functionally conserved than lineage-specific TFBS, even though this comparison dataset is not ideal. See Supplementary Results in Text S1 for details. (TIF) [file pcbi.1003771.s004.tif]

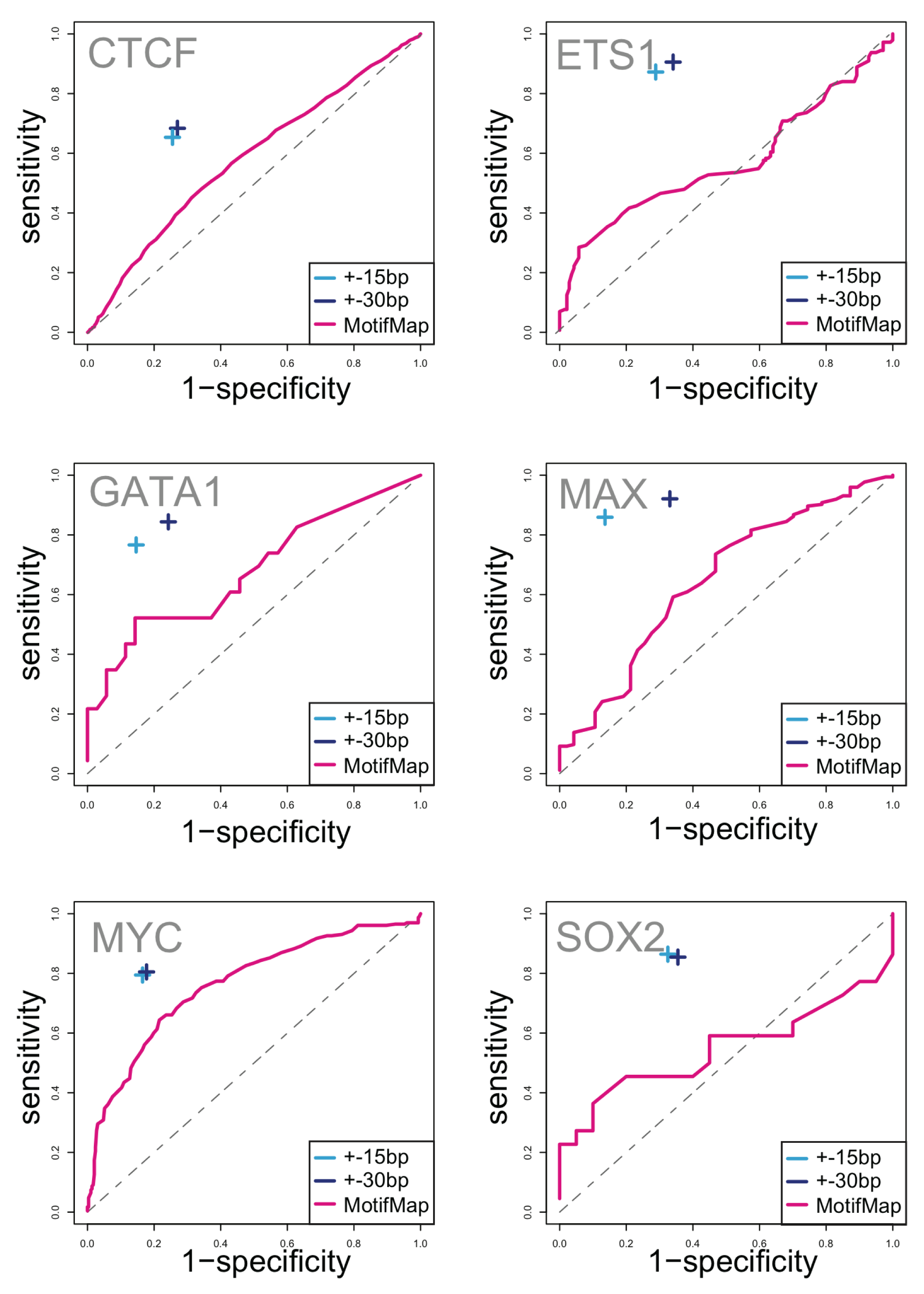

Supplement: Figure S5 — Comparison between our method and MotifMap. A receiver operating characteristic (ROC) curve shows the prediction power between our method and MotifMap. ROC curves for MotifMap were generated using different BBLS thresholds (ranging from zero to the maximum possible BBLS score here, 4.73) to call a TFBS as a conserved one. In our method, we tested two shift sizes, +/−15 bp (light blue) and +/−30 bp (dark blue). The results from MotifMap were based on +/−15 bp shift size (magenta). See Supplementary Results in Text S1 for detailed explanation of the comparison method and how the benchmark dataset was constructed. (TIF) [file pcbi.1003771.s005.tif]

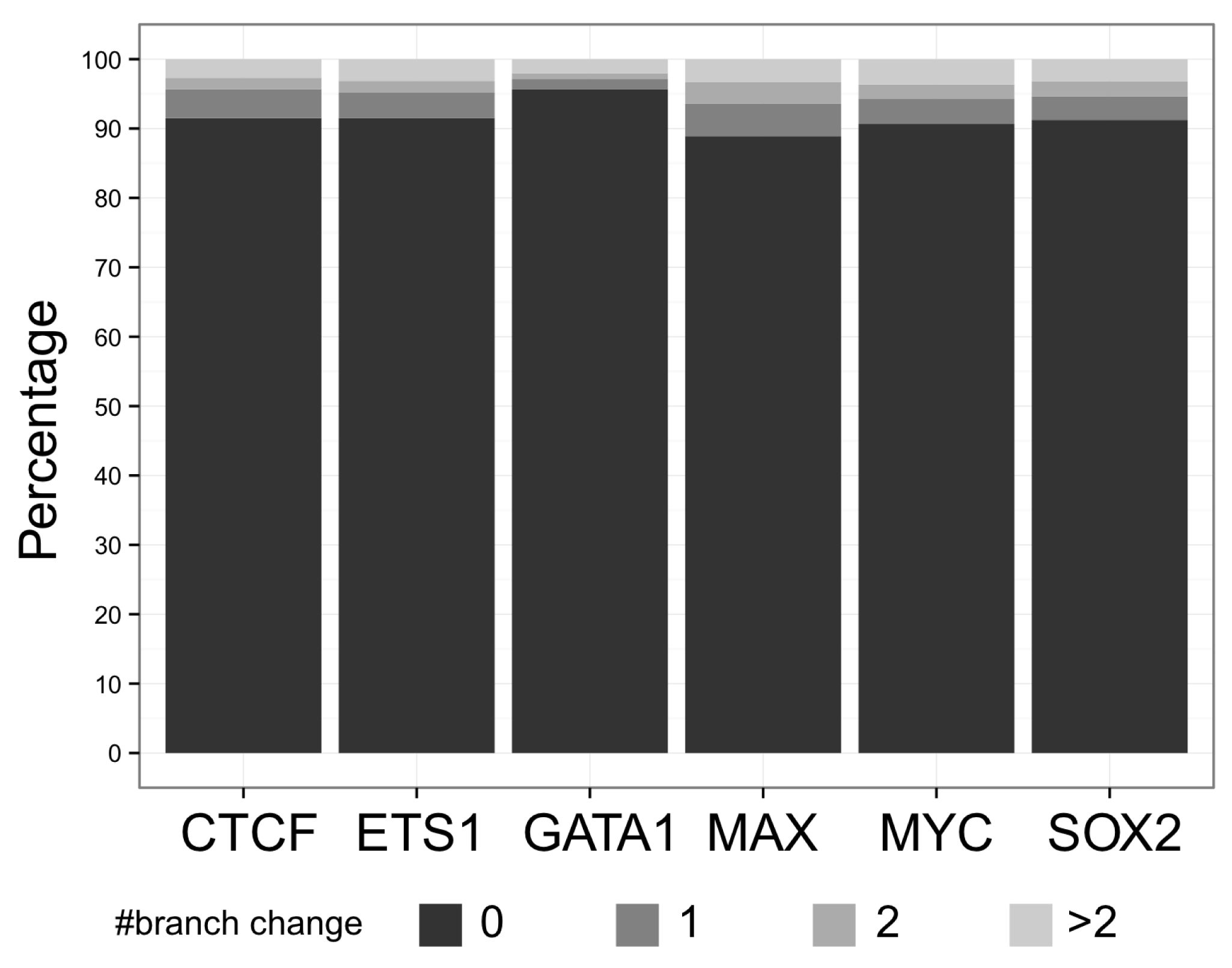

Supplement: Figure S6 — Sensitivity of our method when we add noisy sites in leaf nodes. Sensitivity of our method on the uncertainty of number of binding sites in leaf nodes was determined by randomly deleting/adding 5% sites in +/−100 bp of peak summit in all the species except human. Predictions were compared with original results and the changes of branch of origin for each binding site were counted. Y-axis shows the percentage of binding sites with various branch change. 0 means the prediction of branch of origins remain same before and after we randomly delete or insert binding sites. (TIF) [file pcbi.1003771.s006.tif]

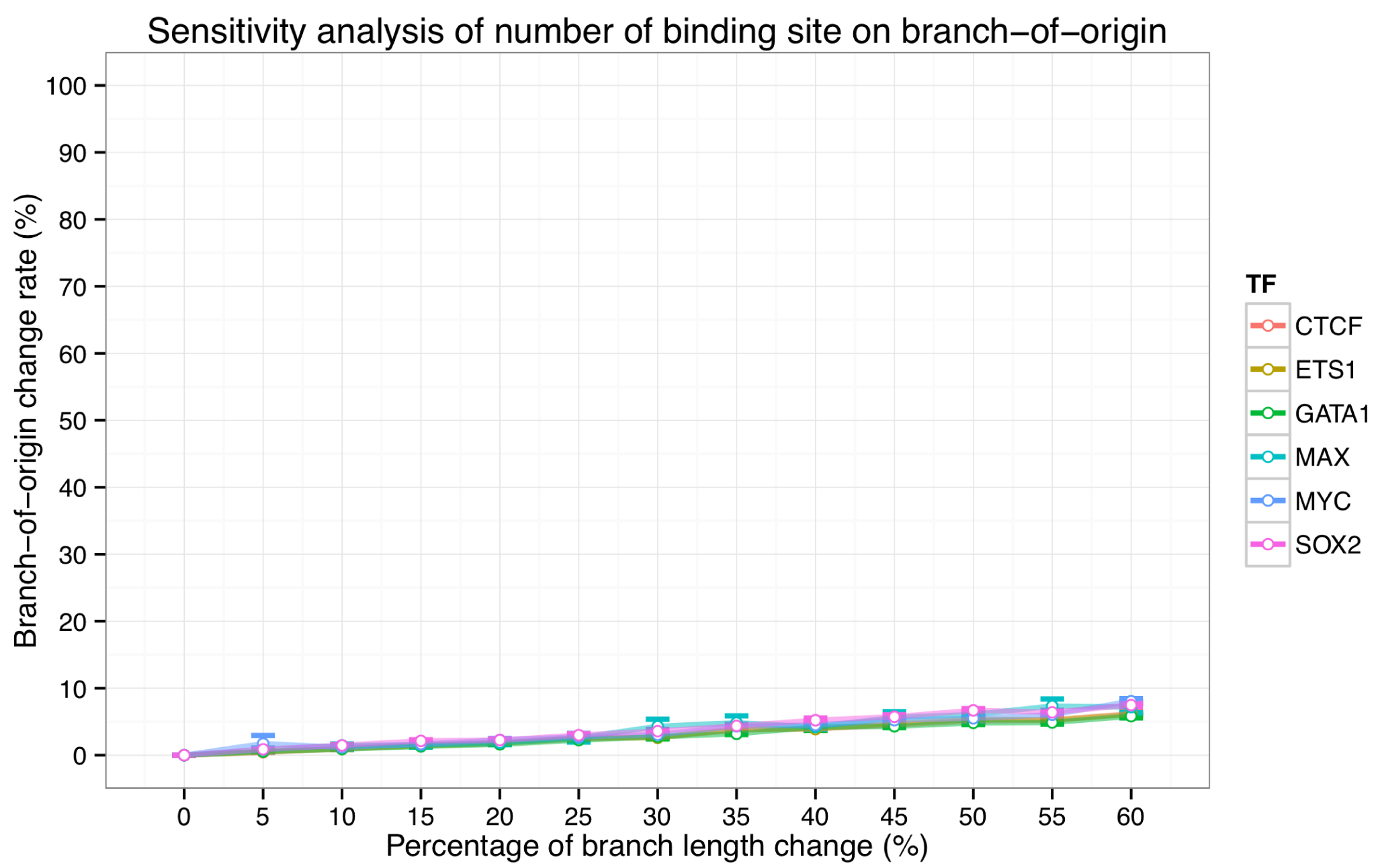

Supplement: Figure S7 — Sensitivity of our method when we change the branch lengths. Sensitivity of our method on the branch length of phylogenetic tree was characterized by randomly changing the branch lengths to a certain extent. The length of each branch in phylogenetic tree can be varied in certain range relative to its original length (shown on X-axis). Y-axis shows the percentage of binding sites that have different branch of origin before and after we randomly change branch lengths in the phylogenetic tree. (TIF) [file pcbi.1003771.s007.tif]
