# Supplementary material for: Tracing the Evolution of Lineage-Specific Transcription Factor Binding Sites in a Birth-Death Framework
Source: PLoS Comput Biol. 2014 Aug 21;10(8):e1003771. doi: 10.1371/journal.pcbi.1003771 (PMC4140645; doi:10.1371/journal.pcbi.1003771)
Supplement: Table S1 — Comparison of consensus motifs. (PDF) [file pcbi.1003771.s008.pdf]

Table S1. Comparison of consensus motifs

TF	PWM in our method$^{\alpha}$	PWM from MEME$^{\beta}$	JASPAR$^{\delta}$	# of sites in our method$^{\varepsilon}$	# of sites from MEME$^{\zeta}$	%
CTCF	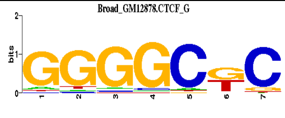	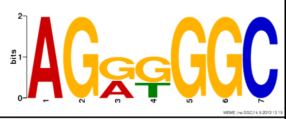	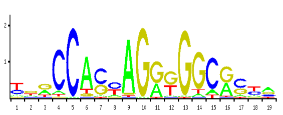	26,586	21,019	28.3%
ETS1	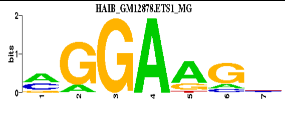	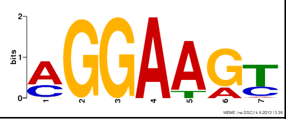	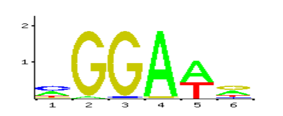	14,883	15,782	72.2%
GATA1	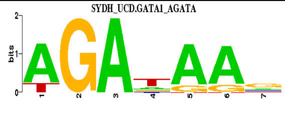	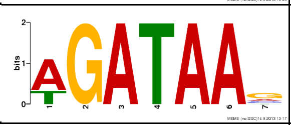	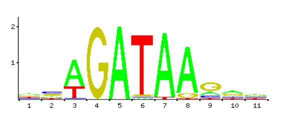	32,348	30,209	75.2%
MAX	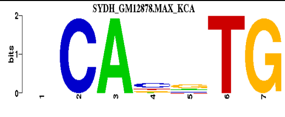	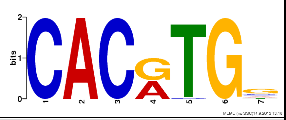	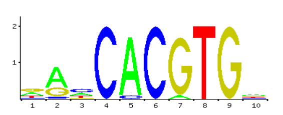	8,935	12,713	83.6%
MYC	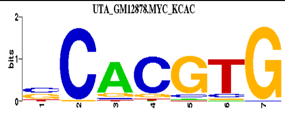		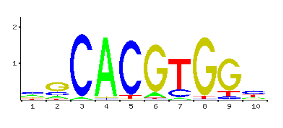	5,274	6,737	68.5%
SOX2	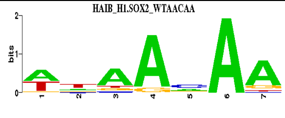		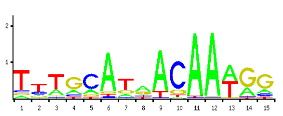	38,530	31,499	30.4%

α: The PWM found within 100bp up-/down-stream of peak summit using our methods;

β: The PWM found by MEME in randomly selected 2,000 200bp peak regions centered on summit;

δ: The PWM in the JASPAR database;

ε: The number of binding site found by our methods;

ζ: The number of binding site found by MEME-FIMO pipeline (note that the PWMs of MYC and SOX2 are from the JASPAR database). The p-value cutoff used in FIMO is 1e-3.

The predicted PWMs were compared with motifs found using the MEME pipeline. Note that MYC and SOX2 motifs cannot be found by MEME perhaps because the percentage of peaks containing consensus binding motif is low. The last column shows the overlapping rate between our prediction and results from MEME (for all except MYC and SOX2) or JASPAR database (only for MYC and SOX2).
