# Supplementary material for: Tracing the Evolution of Lineage-Specific Transcription Factor Binding Sites in a Birth-Death Framework
Source: PLoS Comput Biol. 2014 Aug 21;10(8):e1003771. doi: 10.1371/journal.pcbi.1003771 (PMC4140645; doi:10.1371/journal.pcbi.1003771)
Supplement: Table S2 — Gene functions and pathways associated with hominid-specific TFBS. (PDF) [file pcbi.1003771.s009.pdf]

Table S2. Gene functions and pathways associated with hominid-specific TFBS

Factor	Biological Process	P-val	Fold	Biological pathways	P-val
CTCF	Positive regulation of actin filament polymerization (32 genes)	3e-14	3.45x	Olfactory Signaling Pathway (346 genes)	7e-5
	Retrograde transport, endosome to Golgi (29 genes)	2e-12	3.50x	Phase II conjugation (37 genes)	4e-3
	Positive regulation of protein polymerization (44 genes)	2e-8	2.41x	stearate biosynthesis I (animals) (12 genes)	8e-3
	Detection of chemical stimulus involved in sensory perception of smell (385 genes)	5e-8	2.20x	Regulation of Lipid Metabolism by Peroxisome proliferator-activated receptor alpha (PPARalpha) (32 genes)	1e-2
GATA1	Inositol phosphate metabolic process (16 genes)	2e-4	2.40x	Ketone body metabolism (5 genes)	6e-4
	Positive regulation of histone acetylation (12 genes)	6e-4	2.90x	Mevalonate pathway I (10 genes)	6e-4
	Axon extension involved in axon guidance (14 genes)	1e-3	5.23x	Transmission across Electrical Synapses (5 genes)	2e-3
	Organophosphate catabolic process (11 genes)	2e-3	2.77x	Tryptophan degradation III (eukaryotic) (9 genes)	5e-3
MYC	Synapse assembly (41 genes)	2e-5	3.33x	Olfactor Signaling Pathway (346 genes)	4e-5
	Sensory perception of chemical stimulus (456 genes)	7e-5	2.11x	Neurotransmitter Receptor Binding And Downstream Transmission In The Postsynaptic Cell (89 genes)	6e-4
	Receptor clustering (17 genes)	7e-5	3.33x	NCAM1 interactions (23 genes)	2e-3
	Neuron maturation (25 genes)	1e-4	3.00x	CREB phosphorylation through the activation of Ras (29 genes)	2e-3
SOX2	rRNA processing (109 genes)	2e-4	2.01x	Signaling by Aurora kinases (98 genes)	2e-4
	Detection of stimulus involved in sensory perception (435 genes)	5e-4	1.91x	Aurora B signaling (41 genes)	2e-4
	GTP metabolic process (249 genes)	8e-4	1.50x	The citric acid (TCA) cycle and respiratory electron transport (106 genes)	2e-4
	tRNA modification (22 genes)	1e-3	3.81x	Eukaryotic Translation Elongation (88 genes)	5e-4
ETS1	Ventral spinal cord development (26 genes)	2e-4	3.55x	Mitotic Spindle Checkpoint (19 genes)	4e-4
	Receptor guanylyl cyclase signaling pathway (11 genes)	2e-3	4.51x	APC-Cdc20 mediated degradation of Nek2A (23 genes)	8e-4
	Cell differentiation in spinal cord (37 genes)	3e-3	2.70x	Phosphorylation of Emi1 (20 genes)	6e-3
	Behavioral fear response (15 genes)	4e-3	2.80x	Tetrahydrobiopterin (BH4) synthesis, recycling, salvage and regulation (12 genes)	1e-2
MAX	Establishment of organelle localization (100 genes)	1e-3	3.36x	Signal amplification (16 genes)	5e-3
	Neural crest cell differentiation (57 genes)	3e-3	2.14x	Thrombin signaling through proteinase activated receptors (PARs) (17 genes)	5e-3
	Neural crest cell development (50 genes)	7e-3	2.06x	PAR4-mediated thrombin signaling events (15 genes)	1e-2
	Positive regulation of lipid transport (19 genes)	8e-3	2.20x	Signaling by Robo receptor (23 genes)	1e-2

Shown are the top-ranking biological processes and gene pathways for genes associated with hominid-specific binding sites for each TF. Functional category enrichment was determined relative to the target genes for the comprehensive list of binding sites, with P-values and fold-enrichment over this background set of target genes determined by GREAT.
