# Supplementary material for: Tracing the Evolution of Lineage-Specific Transcription Factor Binding Sites in a Birth-Death Framework
Source: PLoS Comput Biol. 2014 Aug 21;10(8):e1003771. doi: 10.1371/journal.pcbi.1003771 (PMC4140645; doi:10.1371/journal.pcbi.1003771)
Supplement: Table S3 — Performance comparison with MotifMap and PReMod. (PDF) [file pcbi.1003771.s010.pdf]

Table S3: Performance comparison with MotifMap and PReMod

a) Results from +/-15bp shift size in our method

	Sensitivity			Specificity			Accuracy		
	Our method	MotifMap	PReMod	Our method	MotifMap	PReMod	Our method	MotifMap	PReMod
CTCF	**0.6533**	0.3115		0.7405	**0.794**		**0.71**	0.512	
ETS1	**0.8722**	0.5903	0.3633	**0.7074**	0.3669	0.7005	**0.7381**	0.4806	0.4989
GATA1	**0.7664**	0.4348	0.4054	0.8496	**0.8857**	0.6974	**0.8464**	0.7069	0.5959
MAX	**0.8591**	0.4368	0.5202	**0.8596**	0.7447	0.6957	**0.8595**	0.5023	0.5758
MYC	0.7944	**0.8478**	0.3448	**0.8304**	0.4658	0.7802	**0.8215**	0.6341	0.5362
SOX2	**0.8643**	0.0909		0.6708	**1**		**0.6783**	0.5238	

b) Results from +/-30bp shift size in our method

	Sensitivity			Specificity			Accuracy		
	Our method	MotifMap	PReMod	Our method	MotifMap	PReMod	Our method	MotifMap	PReMod
CTCF	**0.6836**	0.3115		0.7257	**0.794**		**0.711**	0.512	
ETS1	**0.9055**	0.5903	0.3633	**0.655**	0.3669	0.7005	**0.7017**	0.4806	0.4989
GATA1	**0.8437**	0.4348	0.4054	0.7529	**0.8857**	0.6974	**0.7565**	0.7069	0.5959
MAX	**0.9208**	0.4368	0.5202	0.6649	**0.7447**	0.6957	**0.6934**	0.5023	0.5758
MYC	0.8049	**0.8478**	0.3448	**0.8184**	0.4658	0.7802	**0.8151**	0.6341	0.5362
SOX2	**0.8543**	0.0909		0.6407	**1**		**0.649**	0.5238	

We compared our method with phylogenetic footprinting methods MotifMap for element level comparison and PReMod for module level comparison (see Supplementary Results in Text S1 for detailed methods used). For MotifMap, BBLS score cutoff to call a conserved TFBS is 1.91 in this table. Bold numbers indicate the best among the three methods. PReMod database does not contain results for CTCF and SOX2. Here we tested our method using two window sizes: +/-15bp and +/-30bp. The shift size in MotifMap is +/-15bp. The overall accuracy (which balances sensitivity and specificity) of our method is much better than MotifMap and PReMod. We also show the comparison between our method and MotifMap using different BBLS thresholds and for all TFs our method outperformed MotifMap (Figure S5).
