# Supplementary material for: Tracing the Evolution of Lineage-Specific Transcription Factor Binding Sites in a Birth-Death Framework
Source: PLoS Comput Biol. 2014 Aug 21;10(8):e1003771. doi: 10.1371/journal.pcbi.1003771 (PMC4140645; doi:10.1371/journal.pcbi.1003771)
Supplement: Table S4 — Gene functions and pathways associated with simian-specific TFBS. (PDF) [file pcbi.1003771.s011.pdf]

Table S4. Gene functions and pathways associated with simian-specific TFBS

Factor	Biological Process	P-val	Fold	Biological pathways	P-val
CTCF	Purine ribonucleoside monophosphate biosynthetic process (20 genes)	5.9e-4	2.12x	Unwinding of RNA (11 genes)	4e-4
	Protein O-linked glycosylation (27 genes)	3e-3	2.05x	Purine nucleotides de novo biosynthesis (11 genes)	4e-4
				Acetylcholine neurotransmitter release cycle (11 genes)	4e-3
				Norepinephrine neurotransmitter release cycle (11 genes)	4e-3
GATA1	Replicative senescence (9 genes)	6e--3	2.05x		
MYC	Negative regulation of insulin receptor signaling pathway (18 genes)	1e-3	2.33x	Platelet calcium homeostasis (21 genes)	9e-3
	Neural precursor cell proliferation (42 genes)	1e-3	2.59x		
	Epithelial cell differentiation involved in prostate gland development (14 genes)	3e-3	2.59x		
	Cell proliferation in forebrain (21 genes)	3e-3	2.59x		
SOX2	Vasoconstriction (21 genes)	2e-4	2.08x	Glutathione redox relations (9 genes)	5e-3
	Establishment of protein localization in Golgi (17 genes)	5e-4	2.18x	Arf1 pathway (20 genes)	5e-3
	Vascular smooth muscle contraction (10 genes)	8e-4	2.18x		
	Protein targeting to Golgi (16 genes)	1e-3	2.18x		
ETS1	Cellular amide metabolic process (20 genes)	3e-3	2.33x	Pyruvate metabolism (10 genes)	8e-3
	Negative regulation of viral genome replication (17 genes)	2e-3	4.51x	Regulation of pyruvate dehydrogenase (PDH) complex (9 genes)	8e-3
	Spermatid nucleus differentiation (11 genes)	3e-3	2.70x	Visual signal transduction: Rods (24 genes)	1e-2
MAX	Positive regulation of phosphatase activity (12 genes)	1e-2	2.12x	Phenylalanine and tyrosine catabolism (13 genes)	1e-2
	Diterpenoid metabolic process (34 genes)	1e-2	2.12x	D-myo-inositol (1,4,5)-triphosphate degradation (20 genes)	1e-2
	Apical protein localization (10 genes)	1e-2	2.12x		
