# Supplementary material for: Tracing the Evolution of Lineage-Specific Transcription Factor Binding Sites in a Birth-Death Framework
Source: PLoS Comput Biol. 2014 Aug 21;10(8):e1003771. doi: 10.1371/journal.pcbi.1003771 (PMC4140645; doi:10.1371/journal.pcbi.1003771)
Supplement: Table S5 — Gene functions and pathways associated with ancestral TFBS. (PDF) [file pcbi.1003771.s012.pdf]

Table S5. Gene functions and pathways associated with ancestral TFBS

Factor	Biological Process	P-val	Fold	Biological pathways	P-val
CTCF	Positive regulation of branching involved ureteric bud morphogenesis (19 genes)	9e-9	2.05x	Glycine betaine degradation (9 genes)	1e-5
	Renal vesicle development (13 genes)	7e-8	2.21x	Folate transformations (9 genes)	5e-4
	Hindlimb morphogenesis (38 genes)	2e-7	2.03x	Interleukin-6 signaling (8 genes)	7e-4
	Blood vessel endothelial cell migration (19 genes)	2e-7	2.03x	Alpha6 beta4 integrin-ligand interactions (11 genes)	2e-3
GATA1	Mesonephros development (19 genes)	1e-9	2.63x	TRAF6 mediated NF-kb activation (13 genes)	2e-3
	Cardiac ventricle development (67 genes)	6e-4	2.13x		
	Heart valve morphogenesis (17 genes)	4e-8	2.99x		
	Cardiac septum development (12 genes)	8e-8	3.52x		
MYC	Ribosome biogenesis (146 genes)	4e-7	2.11x	HIF-a-alpha transcription factor network (80 genes)	6e-6
	Circadian rhythm (66 genes)	2e-6	2.90x	BMAL1: CLOCK/NPAS2 activates gene expression (23 genes)	5e-5
	Regulation of transcription from RNA polymerase II promoter by nuclear hormone receptor (61genes)	3e-4	2.04x	Circadian Clock (32 genes)	2e-4
	Cellular response to biotic stimulus (76 genes)	6e-4	2.55x	Coregulation of androgen receptor activity (29 genes)	5e-4
SOX2	Dentate gurus development (9 genes)	2e-7	2.25x	Validated nuclear estrogen receptor beta network (15 genes)	1e-3
	Central nervous system neuron axonogenesis (16 genes)	1e-5	2.71x		
	Nephron tubule morphogenesis (14 genes)	3e-5	2.13x		
	Erythrocyte development (22 genes)	3e-5	2.84x		
ETS1	Inner mitochondrial membrane organization (26 genes)	2e-3	2.03x	N-glycan trimming in the ER and Calnexin/Calreticulin cycle (13 genes)	2e-3
	Peripheral nervous system neuron development (11 genes)	3e-3	2.26x	Advanced glycosylation endproduct receptor signaling (23 genes)	8e-3
				Recruitment of NuMA to mitotic centrosomes (12 genes)	8e-3
MAX	ATP hydrolysis coupled proton transport (31 genes)	4e-5	4.35x	BMAIL1: CLODK/NPAS2 activates gene expression (23 genes)	9e-6
	Isoprenoid biosynthetic process (22 genes)	4e-5	3.27x	Circadian clock (32 genes)	5e-5
	Cellular response to steroid hormone stimulus (50 genes)	5e-5	2.42x	Transferrin endocytosis and recycling (27 genes)	1e-3
	Regulation of receptor biosynthesis process (16 genes)	7e-5	3.33x	Transcription regulation of white adipocyte differentiation (69 genes)	5e-3
