# Supplementary material for: Tracing the Evolution of Lineage-Specific Transcription Factor Binding Sites in a Birth-Death Framework
Source: PLoS Comput Biol. 2014 Aug 21;10(8):e1003771. doi: 10.1371/journal.pcbi.1003771 (PMC4140645; doi:10.1371/journal.pcbi.1003771)
Supplement: Text S1 — Supplementary methods and supplementary results. (PDF) [file pcbi.1003771.s013.pdf]

Text S1. Supplementary Methods and Supplementary Results

Supplementary Methods

Data preparation

ChIP-seq binding data was obtained for six factors in human and mouse for analogous cell-types across species: GATA1 (PBDE/Erythroblast cell-lines), CTCF, ETS1, MYC, MAX (GM12878/CH12 cell-lines), and SOX2 (E14/H1 cell-lines) [1-4]. We mapped the Illumina sequencing reads (in FASTQ-format) to the human (hg19) and mouse (mm9) genomes using Bowtie [5], using parameters 'bowtie –v 2 –m 1'. We then used the MACS peak caller [6] to calculate to determine enriched regions of high peak density for each ChIP experiment. Parameters used for the MACS peak caller were 'macs14 –g hs –p 1e-4'.

We then obtained orthologous sequences across 46 vertebrate species genome-wide corresponding to ChIP-seq peaks in humans using the 46-way multiz alignments [7] available at the UCSC Genome Browser [8]. To obtain accurate parameter estimates, we used the total data using the complete 46-way alignments to estimate α and β. To determine the binding motif of each factor and to determine the branch of origin for each TFBS, we used the (-100,+100) region window relative to the summit of the peak in humans.

Motif prediction

Binding motifs were predicted using increased branch-specific evolutionary rates using the likelihood ratio test, as mentioned in the main text. To predict motifs with an increased birth rates ancestral to humans among the sequences bound by each factor in humans, we scanned for lineage-specific differences in evolutionary rates by applying our model to the sequence orthologs across all 46 vertebrate species corresponding to protein-bound regions in humans. The model thus detects changes in evolutionary rates along individual branches of the tree, where branch lengths within the phylogeny were taken from [9].

Since the minimum length of most motifs in the JASPAR Core Database is approximately 7bp [10], we tested for increased lineage-specific evolutionary rates across 7-mer seeds. This produced a P-value corresponding to each branch of the phylogeny, reflecting the deviation in birth-death rates of the 7-mer along this branch. To circumvent an exhaustive search across the comprehensive list of all

7-mer motifs, we limited our motif scan to the 1500 most frequently occurring 7-mers in each data set, which was generally the number of 7-mers exhibiting statistically significant overrepresentation within the sequences. We then clustered predicted 7-mer motifs (P<1e-10, according to the likelihood ratio test) according to sequence similarity, condensing each cluster into a single consensus motif as described below, with additional steps to construct position weight-matrix for each TF.

Similar 7-mer motifs predicted along the same branch were clustered iteratively, considering each 7-mer in rank order according to P-value. As many 7-mers were predicted under the threshold for more than one branch, we filtered redundant clusters containing an identical 7-mer, keeping the cluster producing the highest P-value. Only clusters predicted along a branch ancestral to humans (comprising the majority of predictions) were then included in the analyses. At each step, a 7-mer was placed in an existing cluster if it was similar to another 7-mer within the cluster, matching at six of the seven columns (i.e., allowing one mismatch if the two 7-mers were aligned at all seven nucleotide sites, or allowing a single nucleotide offset in the 7-mer alignment with no mismatches). Each cluster thus comprised a set of aligned 7-mers, which was condensed into a single consensus sequence. To emphasize the most significant 7-mer predictions within each cluster, each 7-mer was weighted according to significance of the P-value, with a weight of $W = -\log(p)$ for a 7-mer predicted at a P-value of p. Columns containing less than 15% of the total number of 7-mers in the cluster were removed. Consensus sequences were then generated using criteria similar to [11,12], where each column was assigned single residue if it comprised at least 50% of the total score and at least twice the score of every other nucleotide. In the remaining cases, double nucleotide degeneracy was assigned to sites in which at least 75% of the total score was attributed to two nucleotides, otherwise the site was considered fully-degenerate.

Constructing Position Weight Matrix (PWM) for consensus motif

From the consensus sequences generated from the previous step, PWMs were generated using an iterative approach in which the initial consensus sequence was converted to a PWM. This generates a list of k-mer motifs (k can vary for different TF), and motifs are allowed to be on either strand. Based on this list, an initial PWM can be constructed using the observed data within the (-100,+100) window surrounding all ChIP-seq peaks. A new cutoff is then set, using a score:

$$S = \log\left(\frac{\prod_{i=1}^{k} c_j(w_i)}{\prod_{i=1}^{k} b(w_i)}\right) \tag{1}$$

where $c_j(w_i)$ is the frequency of nucleotide w_i in a given k-mer $w = w_1 w_2 \ldots w_k$ that is a potential binding site, and $b(w_i)$ is the background frequency of nucleotide w_i.

This score represents the quality of the match of the potential k-mer over the expected match. The threshold is set so that, with each iteration, the score of the lowest-ranking k-mer is equal-to or above that of the new motif. In other words, we want to include a large number of peaks using a small number of k-mers. PWMs are updated after each iteration using this approach, until the list of k-mers no longer changes between successive iterations. The final PWM is then used to generate a final list of k-mers. The threshold is set to the score at which the number of peaks containing a binding site increases less than expected from a random list of k-mers, and also includes at least 60% of the total number of peaks. The expected number of peaks containing a specific k-mer is calculated from the background frequencies of the nucleotides in that k-mer. For any given site, the probability at that site, *p*, is the product across each of the columns. If *m* is the width of the peak, then the probability that a peak will contain the k-mer is $P = 1-(1-p)^m$. This gives the expected number of peaks containing each k-mer.

Assessing branch-specific deviations in birth-death rates

To determine motifs that exhibit branch-specific differences in birth-death rates along a specific lineage, we use a likelihood-ratio test. Namely, we compare the total log-likelihood $L(D;\theta_0)$ of the data according to the null model $\theta_0 = [\alpha, \beta]$, in which birth-death rates α and β are constant throughout the phylogeny, to the log-likelihood $L(D;\theta_A)$ of an alternative model $\theta_A = [\alpha, \beta, \alpha_A, \beta_A]$, in which birth-death rates α_A and β_A vary along a single branch relative to the rest of the phylogeny. For both models we estimate the parameters θ_0 and θ_A according to maximum-likelihood as described in the main text. Our framework meets regularity conditions, and thus the scaled deviance

$$2\left[L(D;\theta_A) - L(D;\theta_0)\right] \sim \chi^2 \qquad (2)$$

follows a chi-squared distribution with $|\theta_A| - |\theta_0|$ degrees of freedom [13]. P-values representing the statistical significance of lineage-specific acceleration along a specific branch can thus be determined using an F-test.

Age distributions, repeat-sequence origins, common SNP overlap, and target gene analyses

For each ChIP-seq peak, we determined the most likely ancestral state at each node using the number of TFBSs in each region across species. Motif occurrences included all occurrences of the binding consensus within the region. The origins of human binding sites were thus traced back either to an ancestral lineage within primates (where the number of sites was inferred to decrease along a specific lineage according to the most likely ancestral state), or else was considered 'ancestral' if the motif was inferred to be present in the human-mouse common ancestor.

To determine the overlap of each binding site with transposable elements, we compared the genomic location of each binding site with a known repeat sequence documented by RepeatMasker [14]. We used a simple binomial test to determine enrichment for specific repeat families, using total frequency (p) of each repeat family across the total genome-wide coverage of known repeats. Given the total number of binding sites (N) overlapping known repeats, the number of TFBSs overlapping each repeat family then follows a binomial distribution with probability of success p and number of trials N ; P-values representing the enrichment for each repeat family can be calculated using this distribution.

Trends between human variation and branch-of-origin of TFBSs were conducted using common SNP data from the dbSNP 137 track [15] available at the UCSC Table Browser [8]. To rule out the bias introduced by background SNP density, we did the following analysis. For those TFBSs (grouped into different branches of origin) overlapping with common SNPs, we extended 1kb to both directions (i.e. 2k window) and counted the number of common SNPs in this 2kb window. We also tested window size of 1kb and 250bp. We found that there are no significant differences of SNP density surrounding the TFBSs with different branches of origin (Figure S3).

To determine functional enrichment of genes close to newly-derived binding sites, we used GREAT [16] to determine category enrichments for biological processes in Gene Ontology (GO) [17] as well as pathways in PANTHER and Pathway Commons [18,19]. Hominid-specific TFBSs were assigned to the single closest gene, up to 100kb of the nearest TSS (Table S2). We also tested Simian-specific TFBS and ancestral ones (Table S3 and Table S4). We used the complete list of binding sites for each factor as the background set, and thus all category enrichments are shown relative to the full list of target genes for each factor.

Supplementary Results

TFBS origins show varying contributions of transposable element derived sequences

We assessed the amount of overlap between newly-derived TFBSs and annotated TEs determined by RepeatMasker [14]. Consistent with previous studies, a substantial fraction of binding sites with recent origin were located within TEs. The fraction of TFBSs overlapping with documented TE sequences is shown in Figure S1 for each TF according to the branch of origin of the binding sites. The number of TE-derived sites varied between factors. P-values of enrichment for TEs among hominid-specific binding sites were consistently under $P<1e-7$ for all factors (Supplementary Methods).

The fraction of TFBSs derived from TEs remained relatively constant throughout recent primate evolution, with a decreased overlap with documented TEs in older TFBSs. This may be unexpected, as the propagation of specific TE family is often lineage-specific [20,21]. Since many TE families are likely to propagate along some specific branches of the phylogeny, we tested whether TE-derived TFBSs were associated with different TE families with different times of origin. GATA1 binding sites have been shown to be associated with LINE and LTR families [22], and indeed we found the high enrichment among L1, L2, and LTR/ERV1 family [23]. However, some enrichment was age-specific. For example, for LTR/ERV1 and GATA1 sites, only sites appearing near the origin of recent primates exhibiting enrichment for ERV1. Sites originating earlier in primate history were more associated with MIR. The most recently-derived GATA1 binding sites were also often associated with *Alu*, although fewer of the oldest binding sites were found within *Alu* elements. These results are consistent with the recent *Alu* expansion in hominids, preceded by LTR and then by MIR expansion earlier in primate history [24,25]. Similar age-specific enrichments were observed for SOX2. We found that *Alu* and ERV1 among the most recently derived binding sites. In the case of CTCF, there was a notable rise in TE-derived sites among TFBSs originating specifically along the branch of the most recent hominid ancestor. This was found to be largely due to members of the ERVK family. CTCF appeared to have shorter time of expansion due to TE propagation, despite a general overall trend for older TFBSs to have less overlap with documented TEs in older primate lineages or those shared with mouse. This may suggest some regulatory innovation through the use of TE-derived TFBSs specifically along this branch, more consistent with previous reports of lineage-specific use of TE-derived TFBSs in mammals [26]. Human CTCF binding sites were also associated with ERV1, ERVL, and L1, which is consistent with previous findings [27], although again these enrichments were found to be largely age-specific.

Comparison with the VISTA enhancer database

To further evaluate our lineage-specific TFBS prediction, we intersected our prediction with enhancers from the VISTA Enhancer Browser [28]. Information for 1,601 human VISTA enhancers was retrieved. Among these 1,601 enhancers, 206 enhancers overlapped with at least one TFBS in our six TFs dataset. We then divided these 206 enhancers into three categories based the percentage of ancestral TFBS (before human mouse split) within each enhancer. If the percentage of ancestral TFBS exceeds 50%, that enhancer is defined as 'more ancestral' (141 in total). If the percentage of ancestral TFBS is below 50%, that enhancer is defined as 'more lineage-specific' (39 in total). Otherwise, the enhancer is marked as 'neutral' (26 in total). In Figure S4, we show the percentage of enhancers that also show enhancer activity in mouse (defined as positive) for three different categories we defined. Overall, 'more ancestral enhancer' has higher positive enhancer rate as compared to 'more lineage-specific enhancers' or 'neutral enhancer', which is generally consistent with our computational prediction that the ancestral TFBS are more functionally conserved. However, we note that this comparison dataset is not perfect because the size distribution of enhancers (from 314bp to 8,061bp) is much larger than the size of TFBS. It is possible that there are other TFBS (within the enhancers) not included in our six TFs. This may explain why there are still a quite large proportion of 'more lineage-specific' enhancers have enhancer activity in mouse.

Comparison with the methods using phylogenetic footprinting approaches

We evaluated the performance of our method in predicting the age of TFBS as compared to traditional methods based on phylogenetic footprinting approaches. We compared with phylogenetic footprinting methods at both element level (using MotifMap [29]) and module level (using PReMod [30]). Since phylogenetic footprinting approaches only predict if the TFBS is conserved in a phylogeny and do not provide information of specific lineage where the TFBS originated, we conducted our evaluation by comparing the ancestral TFBS (i.e., the ones conserved between human and mouse) predicted by our method with the predictions from MotifMap and the predictions from PReMod. We used two windows sizes, +/-15bp and +/-30bp, in order to directly compare with MotifMap (+/-15bp shift size).

MotifMap contains conserved TFBS identified using Bayesian Branch Length Score (BBLS) [29]. BBLS and its previous version Branch Length Score (BLS) [31,32] are among the latest algorithms for conserved TFBS discovery. It quantifies the conservation level of TFBS in a given phylogenetic (in this case, the placental mammal multiz alignments). In contrast to MotifMap, PReMod contains 100,000

computational predicted cis-regulatory modules conserved between human and mouse based on statistically significant clusters of phylogenetically conserved TFBS within modules [30].

To make a fair comparison, we first constructed a benchmark data set from the ChIP-seq data in human-mouse analogous cells used in this study. Positive cases (conserved TFBS originated before human-mouse common ancestor) are 200bp regions centered on human ChIP-seq peak summit that satisfy the following requirements: 1) For the same TF, there exists ChIP-seq peak in mouse analogous cells with mouse peak summit within +/-200bp of the human peak summit; and 2) TFBS exist in both human and mouse peak region. Negative cases (TFBS originated after human-mouse common ancestor) are 200bp regions centered on human ChIP-seq peak summits that do not have shared peaks in mouse and do not have TFBS in mouse orthologous region.

For the MotifMap method, we chose 1.91 or 40% BBLS score (equal to 60% confidence level [31,33]) as threshold to call a conserved TFBS. For PReMod method, ancestral regions are defined as regulatory modules shared between human and mouse. Table S3 shows the comparisons of MotifMap, PReMod, and our method. We evaluated sensitivity, specificity, and accuracy (i.e., (true_positive + true_negative)/(total # of cases)) of these three methods. The overall accuracy of our method is much better than MotifMap and PReMod. In addition to results from a fixed threshold for MotifMap used in the Table S3, we also plot the ROC curves when different threshold scores were used in MotifMap (Figure S5). For all TFs, our method outperformed MotifMap.

References

1. Lister R, Pelizzola M, Dowen RH, Hawkins RD, Hon G, et al. (2009) Human DNA methylomes at base resolution show widespread epigenomic differences. Nature 462: 315-322.
2. Mouse EC, Stamatoyannopoulos JA, Snyder M, Hardison R, Ren B, et al. (2012) An encyclopedia of mouse DNA elements (Mouse ENCODE). Genome Biol 13: 418.
3. Myers RM, Stamatoyannopoulos J, Snyder M, Dunham I, Hardison RC, et al. (2011) A user's guide to the encyclopedia of DNA elements (ENCODE). PLoS biology 9: e1001046.
4. Chen X, Xu H, Yuan P, Fang F, Huss M, et al. (2008) Integration of external signaling pathways with the core transcriptional network in embryonic stem cells. Cell 133: 1106-1117.
5. Langmead B, Trapnell C, Pop M, Salzberg SL (2009) Ultrafast and memory-efficient alignment of short DNA sequences to the human genome. Genome Biol 10: R25.
6. Zhang Y, Liu T, Meyer CA, Eeckhoute J, Johnson DS, et al. (2008) Model-based analysis of ChIP-Seq (MACS). Genome Biol 9: R137.
7. Miller W, Rosenbloom K, Hardison RC, Hou M, Taylor J, et al. (2007) 28-way vertebrate alignment and conservation track in the UCSC Genome Browser. Genome Res 17: 1797-1808.
8. Karolchik D, Hinrichs AS, Furey TS, Roskin KM, Sugnet CW, et al. (2004) The UCSC Table Browser data retrieval tool. Nucleic Acids Res 32: D493-496.
9. Hedges SB, Dudley J, Kumar S (2006) TimeTree: a public knowledge-base of divergence times among organisms. Bioinformatics 22: 2971-2972.
10. Sandelin A, Alkema W, Engstrom P, Wasserman WW, Lenhard B (2004) JASPAR: an open-access database for eukaryotic transcription factor binding profiles. Nucleic Acids Res 32: D91-94.
11. Matys V, Fricke E, Geffers R, Gossling E, Haubrock M, et al. (2003) TRANSFAC: transcriptional regulation, from patterns to profiles. Nucleic Acids Res 31: 374-378.
12. Cavener DR (1987) Comparison of the consensus sequence flanking translational start sites in Drosophila and vertebrates. Nucleic Acids Res 15: 1353-1361.
13. Davison AC (2003) Statistical Models. New York: Cambridge University Press.
14. Smit AF, Hubley R, Green P (1996-2010) RepeatMasker Open-3.0.
15. Sherry ST, Ward MH, Kholodov M, Baker J, Phan L, et al. (2001) dbSNP: the NCBI database of genetic variation. Nucleic Acids Res 29: 308-311.
16. McLean CY, Bristor D, Hiller M, Clarke SL, Schaar BT, et al. (2010) GREAT improves functional interpretation of cis-regulatory regions. Nat Biotechnol 28: 495-501.
17. Ashburner M, Ball CA, Blake JA, Botstein D, Butler H, et al. (2000) Gene ontology: tool for the unification of biology. The Gene Ontology Consortium. Nat Genet 25: 25-29.
18. Mi H, Guo N, Kejariwal A, Thomas PD (2007) PANTHER version 6: protein sequence and function evolution data with expanded representation of biological pathways. Nucleic Acids Res 35: D247-252.
19. Cerami EG, Bader GD, Gross BE, Sander C (2006) cPath: open source software for collecting, storing, and querying biological pathways. BMC Bioinformatics 7: 497.
20. Burns KH, Boeke JD (2012) Human transposon tectonics. Cell 149: 740-752.
21. Cordaux R, Hedges DJ, Batzer MA (2004) Retrotransposition of Alu elements: how many sources? Trends Genet 20: 464-467.
22. Chung D, Kuan PF, Li B, Sanalkumar R, Liang K, et al. (2011) Discovering transcription factor binding sites in highly repetitive regions of genomes with multi-read analysis of ChIP-Seq data. PLoS Comput Biol 7: e1002111.
23. Renan MJ, Reeves BR (1987) Chromosomal localization of human endogenous retroviral element ERV1 to 18q22----q23 by in situ hybridization. Cytogenet Cell Genet 44: 167-170.
24. Huda A, Marino-Ramirez L, Jordan IK (2010) Epigenetic histone modifications of human transposable elements: genome defense versus exaptation. Mob DNA 1: 2.

25. Lander ES, Linton LM, Birren B, Nusbaum C, Zody MC, et al. (2001) Initial sequencing and analysis of the human genome. Nature 409: 860-921.
26. Schmidt D, Schwalie PC, Wilson MD, Ballester B, Goncalves A, et al. (2012) Waves of retrotransposon expansion remodel genome organization and CTCF binding in multiple mammalian lineages. Cell 148: 335-348.
27. Kunarso G, Chia NY, Jeyakani J, Hwang C, Lu X, et al. (2010) Transposable elements have rewired the core regulatory network of human embryonic stem cells. Nat Genet 42: 631-634.
28. Visel A, Minovitsky S, Dubchak I, Pennacchio LA (2007) VISTA Enhancer Browser--a database of tissue-specific human enhancers. Nucleic Acids Res 35: D88-92.
29. Xie X, Rigor P, Baldi P (2009) MotifMap: a human genome-wide map of candidate regulatory motif sites. Bioinformatics 25: 167-174.
30. Blanchette M, Bataille AR, Chen X, Poitras C, Laganiere J, et al. (2006) Genome-wide computational prediction of transcriptional regulatory modules reveals new insights into human gene expression. Genome Res 16: 656-668.
31. Stark A, Lin MF, Kheradpour P, Pedersen JS, Parts L, et al. (2007) Discovery of functional elements in 12 Drosophila genomes using evolutionary signatures. Nature 450: 219-232.
32. Xie X, Mikkelsen TS, Gnirke A, Lindblad-Toh K, Kellis M, et al. (2007) Systematic discovery of regulatory motifs in conserved regions of the human genome, including thousands of CTCF insulator sites. Proc Natl Acad Sci U S A 104: 7145-7150.
33. Kheradpour P, Stark A, Roy S, Kellis M (2007) Reliable prediction of regulator targets using 12 Drosophila genomes. Genome Res 17: 1919-1931.
